# Prediction of effector protein structures from fungal phytopathogens enables evolutionary analyses

**DOI:** 10.1038/s41564-022-01287-6

**Published:** 2023-01-05

**Authors:** Kyungyong Seong, Ksenia V. Krasileva

**Affiliations:** Department of Plant and Microbial Biology, University of California, Berkeley, CA USA

**Keywords:** Microbiology, Applied microbiology

## Abstract

Elucidating the similarity and diversity of pathogen effectors is critical to understand their evolution across fungal phytopathogens. However, rapid divergence that diminishes sequence similarities between putatively homologous effectors has largely concealed the roots of effector evolution. Here we modelled the structures of 26,653 secreted proteins from 14 agriculturally important fungal phytopathogens, six non-pathogenic fungi and one oomycete with AlphaFold 2. With 18,000 successfully predicted folds, we performed structure-guided comparative analyses on two aspects of effector evolution: uniquely expanded sequence-unrelated structurally similar (SUSS) effector families and common folds present across the fungal species. Extreme expansion of lineage-specific SUSS effector families was found only in several obligate biotrophs, *Blumeria graminis* and *Puccinia graminis*. The highly expanded effector families were the source of conserved sequence motifs, such as the Y/F/WxC motif. We identified new classes of SUSS effector families that include known virulence factors, such as AvrSr35, AvrSr50 and Tin2. Structural comparisons revealed that the expanded structural folds further diversify through domain duplications and fusion with disordered stretches. Putatively sub- and neo-functionalized SUSS effectors could reconverge on regulation, expanding the functional pools of effectors in the pathogen infection cycle. We also found evidence that many effector families could have originated from ancestral folds conserved across fungi. Collectively, our study highlights diverse effector evolution mechanisms and supports divergent evolution as a major force in driving SUSS effector evolution from ancestral proteins.

## Abstract (web summary)

Evolutionary analysis guided by predicted structures of AlphaFold 2 elucidates novel aspects of rapidly evolving pathogen effectors from fungal phytopathogens.

## Main

Fungal phytopathogens rely on secreted proteins termed effectors to suppress plant immunity, modify host cellular activities and successfully colonize the hosts^1^. However, lack of sequence similarity, functional annotations and commonly shared sequence features of effectors hinders elucidating their evolutionary relationships^2^. Many effectors are unrelated by their primary sequences but share similar structures^3–10^. These effectors are major players in the battlefield of plant immunity and fungal pathogens. Plant intracellular immune receptors can evolve specificity towards these effectors to acquire resistance against the pathogens, as represented with *Magnaporthe oryzae* Avrs and ToxB (MAX) effectors and their cognate immune receptors^11–14^. In turn, pathogens lose and regain effectors to evade immune recognition^15–17^. Such sequence-unrelated structurally similar (SUSS) effectors, the sequence similarity of which cannot be nearly or entirely detected with modern bioinformatics tools despite the structural resemblance, repeatedly appear across phytopathogens, signifying their importance in pathogen evolution. However, only a few classes of fungal SUSS effector families have been discovered so far^3–10^.

Divergent evolution may drive effector evolution^3^. A group of SUSS effectors might have originated from a common ancestor but could have lost detectable sequence similarity through rapid divergence. We proposed computational structural genomics as a framework to reveal such evolutionary connections obscured by sequence dissimilarity^7^. The success of this predicted structure-driven analysis was exemplified by the identification of the MAX effector cluster, which could not be revealed by remote homology searches alone^18^. With the availability of AlphaFold 2 (AF2) (refs. ^19,20^), secretome-wide structure prediction and analysis have provided further insights. For instance, many important effectors from *Fusarium oxysporum* f. sp*. lycopersici* could be grouped into a few structural families, including Fol dual-domain (FOLD) effector family^8^. In *Venturia inaequalis*, MAX effectors represented one of the most expanded families^21^. Such structural analyses have reinforced the divergent evolution hypothesis in that pathogen virulence factors may have evolved through frequent duplications and rapid divergence of common folds.

We proposed that computational structural genomics at a comparative scale would reveal novelty and commonality of effectors and better elucidate effector evolution across diverse species in the fungal kingdom^7^. To elucidate effector evolution at the structural level, we predicted with AF2 the folds of 26,653 secreted proteins from 14 agriculturally important fungal phytopathogens^22^, six non-pathogenic fungi and oomycete *Phytophthora infestans* as an outgroup. In this Resource, we focus on two aspects of effector evolution: uniquely expanded SUSS effector families and common folds present across the fungal species. We highlight how structural information overlaid on sequence-unrelated effectors can provide insights into effector evolution.

## Results

### Structure prediction for fungal secretomes with AF2

**Fig. 1:**
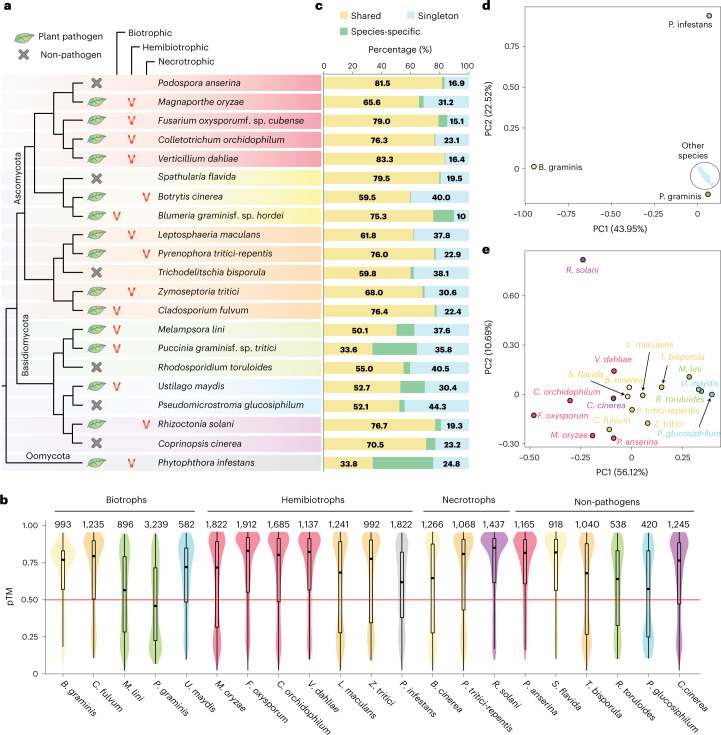
The design of the comparative genomic study and the statistics of structure prediction and secretome clustering. **a**, Cladogram reconstructed on the basis of MycoCosm^55^ and lifestyles of the 21 species included in this study. The plant pathogens are classified as biotrophs, necrotrophs and hemibiotrophs, which undergo both biotrophic and necrotrophic stages. The background highlights reflect phylogenetic classification at the order or subdivision level. **b**, The distribution of pTM scores used to measure the structure prediction quality. The colours of the violin plots reflect those in the phylogeny in **a**. The total number of secreted proteins for each species is indicated on the top of the plots. In the box plot, the bounds of the box represents 25th to 75th percentiles, with a bold line highlighting the median. The whiskers are drawn to the minima and maxima with the length of the whiskers capped at 1.5× the interquartile range. Outlier points are not shown in the box plot. **c**, The proportion of clustered or singleton proteins in whole-secretome clustering. The secretomes of the 21 species are clustered on the basis of sequence and structural similarities. The clusters are categorized as ‘shared’ if the cluster members come from more than one species, and ‘species-specific’ otherwise. If the protein does not form a cluster with at least one other protein, it is ‘singleton’ (Supplementary Table 2). **d**,**e**, PCA on the copy number variations of the clusters in the whole-secretome clustering output with all 21 species included (**d**) and without the three outliers, *Blumeria graminis*, *Puccinia graminis* and *Phytophthora infestans* (**e**). Singletons were not used for the analysis. The species that belong to the same class or subdivision are indicated with the same colour, and the colours correspond to the background highlights given in **a**.

To perform comparative analyses, we predicted with AF2 the structures of 26,653 proteins collected from 21 species’ secretomes (Fig. 1 and Supplementary Table 1). This list of species includes agriculturally important phytopathogens that span across two divisions, Ascomycota and Basidiomycota, with various lifestyles and host ranges^22^. We added a putatively saprotrophic, non-phytopathogenic species per order or subdivision as a control, and the oomycete *P. infestans* as an outgroup for its importance. We used the pTM score provided by AF2 as a global measure of prediction quality. The pTM score of 0.50 was used as a threshold to select reliably predicted folds as in our former study^7^.

In comparison with the structural models that we previously produced with TrRosetta for *M. oryzae*^7^, the estimated precision of AF2 models was typically greater (Supplementary Fig. 1). However, even with the enhanced prediction performance, only 55 additional protein structures were modelled by AF2. Moreover, 612 (33.5%) of *M. oryzae*’s secreted proteins missed by TrRosetta could also not be predicted by AF2 (Supplementary Fig. 2). Overall, AF2 predicted from 47.0% to 81.5% of the secreted proteins across the species in this study (Fig. 1b and Supplementary Table 1). In total, 17,944 (67%) out of 26,653 proteins were modelled with the pTM scores >0.50. The lifestyle of the species was not a determining factor in the performance of AF2. For instance, AF2 predicted approximately 75% of the secreted protein structures from biotrophs, *Blumeria graminis* f. sp. *hordei* (*Bgh*), *Cladosporium fulvum* and *Ustilago maydis* (Fig. 1b and Supplementary Table 1). Conversely, only about 50% of the secreted proteins were predicted for the two biotrophs, *P. graminis* f. sp. *tritici* (*Pgt*) and *Melampsora lini*. Varying performance of AF2 was also observed in species with other lifestyles.

### Secretome clustering with sequence and structural comparison

To reveal evolutionary connections between secreted proteins, we clustered the secretome of individual species on the basis of sequence and structural similarities. The similarity comparisons were performed sequentially for sequence-to-sequence with BLASTP, sequence-to-profile with HHblits, profile-to-profile with HHsearch and structure-to-structure with TM-align as in our previous study^7^. The proportion of clustered proteins ranged from 29.0% in *Pseudomicrostroma glucosiphilum* to 76.2% in *Bgh* (Supplementary Tables 2 and 3). For a comparative analysis, we clustered the entire secretomes of the 21 species used in this study (Supplementary Tables 2 and 4). Overall, 7,207 (27%) out of 26,653 proteins, the majority of which did not have predicted structures, remained as singletons (Supplementary Fig. 3 and Supplementary Table 2). However, 4,087 (15%) proteins initially found as singletons in individual species’ secretome had sequence or structure-related proteins in other species and could be assigned to the clusters (Supplementary Fig. 4). In total, 19,446 (73%) of the secreted proteins had at least one homologue or analogue within or outside the species’ secretome, forming clusters or families. We classified the proteins into ‘shared’ if they belonged to clusters of two or more species and ‘species-specific’ otherwise (Fig. 1c). Among fungal species, only *Bgh*, *M. lini*, *Pgt* and *U. maydis* displayed a relatively high proportion of species-specific secreted proteins (>10%) (Supplementary Table 2). Most proteins, except for the singletons, belonged to shared clusters, possibly suggesting that many proteins might have common ancestral origins.

### Secretome compositions reflect evolutionary relationships

We examined the copy number variations within the clusters with principal component analysis (PCA) to reveal any patterns associated with the clusters (Supplementary Table 5). In the PCA performed on the clusters generated only with sequence-to-sequence comparison with BLASTP, the species were spread largely on the basis of their evolutionary distances (Supplementary Fig. 5). The exceptions were the two obligate biotrophs, *Bgh* and *Pgt*, with relatively large genome sizes and high proportions of transposable elements within the genomes^23,24^. However, the first two principal components could explain only 37.6% of the variance. On the other hand, with the final clusters constructed with sensitive sequence similarity searches and structural comparisons, the first two principal components captured about 66% of the variance (Fig. 1d,e). Still, the distances between the fungal species generally reflected their phylogenetic relationships. This suggested that except for some obligate biotrophs that may undergo distinct evolution, the closer the evolutionary distance is between the species, the more similar the compositions of their secretomes are likely to be. However, even such evolutionary connections may be masked by sequence dissimilarities between related proteins.

### Some pathogens encode species-specific effector families

**Fig. 2:**
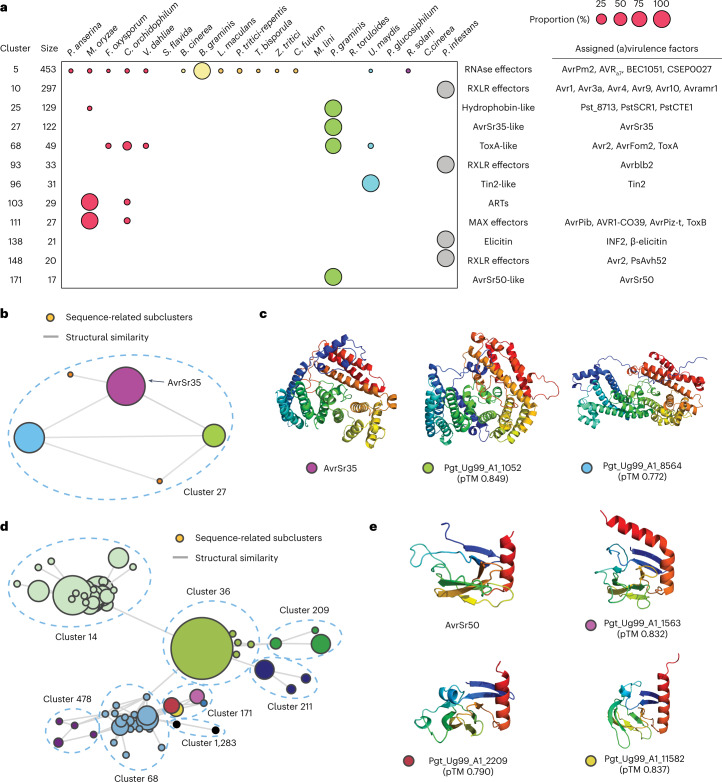
The expanded effector families in phytopathogens and new classes of SUSS effectors. **a**, The nearly or entirely species-specific effector families with putative functions or known virulence factors. The relative compositions of individual species in each cluster are indicated with circles of varying sizes. Only a subset of known virulence factors are indicated (Supplementary Tables 5 and 6). **b**,**d**, The networks of cluster 27 (**b**), as well as cluster 171 and other related clusters (**d**). Each node represents a sequence-related subcluster or singleton, and the edges indicate structural similarity between the subclusters or singletons. The size of the nodes varies, depending on the number of subcluster members. **c**,**e**, The experimentally determined structure of AvrSr35 (PDB: 7XC2) (**c**) (ref. ^26^) and AvrSr50 (PDB: 7MQQ) (**e**) (ref. ^30^) or predicted structures from *Puccinia graminis* selected from the subclusters in clusters 27 and 171. The coloured dots in the labels indicate the membership of the proteins and correspond to those of nodes in **b** and **d**.

We examined nearly (>80%) or entirely species-specific clusters with known virulence factors collected from the literature and the Pathogen-Host Interactions database (Supplementary Tables 5 and 6) (ref. ^25^). Consistent with the observation that *Bgh*, *Pgt* and *P. infestans* were the outliers (Fig. 1d), only these species had nearly or entirely species-specific, highly expanded effector families with 100 or more members (Fig. 2a). There were also other large clusters, such as cluster 29, 31, 40 and 62, nearly exclusive to *Bgh* or *Pgt*; however, no virulence factors related to these clusters have been studied to our knowledge (Supplementary Fig. 6). The Tin2-like effector family in *U. maydis*, as well as MAX effector and ADP-ribosyl transferase (ART) families in *M. oryzae* were among the largest (Fig. 2a). Nonetheless, these families only had about 30 members. In other fungal phytopathogens, we did not observe any nearly or entirely species-specific effector families with comparable sizes (>15 members). This result highlighted the unique evolution of fungal obligate biotrophs, *Bgh* and *Pgt*, with extreme expansions of a few effector families.

### Known virulence factors represent new SUSS effector classes

We examined sequence and structural similarity within the species-specific clusters that include known virulence factors (Fig. 2a). We found that these clusters would represent novel SUSS effector families, as the sequence similarity between the entire members could not be bioinformatically detected while the members share structural similarity. For instance, cluster 27 was composed of three sequence-related subclusters and two singletons connected by structural similarity (Fig. 2b); this cluster included a known avirulence factor, AvrSr35 (ref. ^26^) (Fig. 2c). Cluster 171 was initially interwound with other clusters in a complex manner as a larger network (Fig. 2d), due to partial structural resemblance of the core β-strands (Supplementary Fig. 7)^27–29^. However, our clustering method could properly separate the network into clusters with greater intra-cluster structural similarities. Eventually, cluster 171 had three sequence-related subclusters and a singleton, connected by structural similarity (Fig. 2d). AvrSr50 (ref. ^30^) represented this SUSS effector cluster (Fig. 2e).

We explored the non-secreted protein structures of *Pgt* to evaluate whether AvrSr35 and AvrSr50 folds are unique to the secreted proteins. There were 35 non-secreted proteins predicted to adopt the AvrSr35 fold; yet, they were homologous to the secreted AvrSr35-like proteins at the sequence level (Supplementary Table 7). Sequence-unrelated non-secreted proteins with a similar fold were not found. *AvrSr35* contains seven exons, and its product is 578 amino acids long. Together with widespread transposable elements across the genome and rapid divergence of the members, we speculate that the annotation may not be correct for some genes. On the contrary, the AvrSr50-like fold was absent in the non-secreted proteins (Supplementary Table 7), supporting effector fold enrichment in secretome. Together, the analysis suggested that SUSS effectors have repeatedly evolved in phytopathogens’ secretome, and known virulence factors may represent the novel SUSS effector families.

### Extreme SUSS effector expansion results in common motifs

The largest SUSS effector cluster was the RNAse-like effector family, composed of 453 members (Fig. 2a). Although many fungal species had a few RNAse-like proteins, 426 members were from *Bgh*, representing 43% of the *Bgh*’s secretome. Furthermore, the RNAse-like fold was absent in the sequence-unrelated non-secreted *Bgh* proteins (Supplementary Table 7), supporting the fold’s specialization in pathogenesis. Examining branch-level selection pressure on *Bgh*’s RNAse-like effectors, we found that the extreme expansion and divergence of the family members have been driven by mixed evolutionary mechanisms, including diversifying selection and relaxed purifying selection at some terminal branches relative to the rest of the phylogeny (Supplementary Fig. 8).

**Fig. 3:**
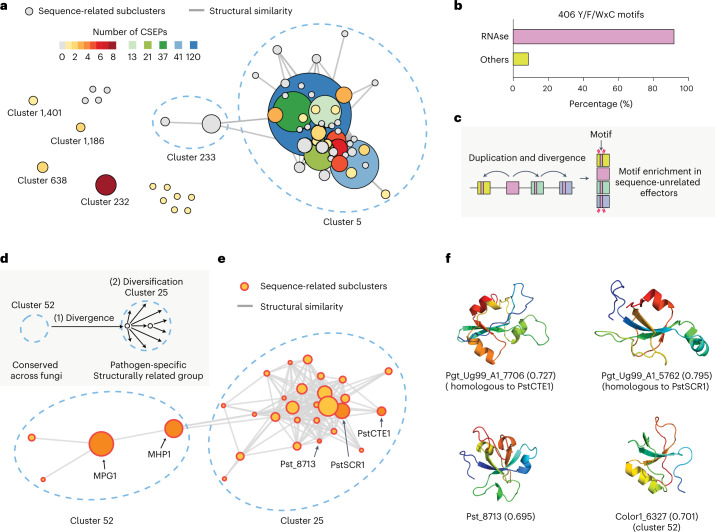
Evolution of RNAse effector family *Blumeria graminis* and hydrophobin-like effector family in *Puccinia graminis* with extreme expansion and divergence. **a**, The network of the RNAse supercluster. Each node represents a sequence-related subcluster or singleton, and the edges indicate structural similarity between the subclusters or singletons. The size of the nodes varies on the basis of the number of members. The subcluster is coloured to indicate the number of CSEPs. Clusters or singletons, other than clusters 5 and 233, were retrieved into the supercluster by lowering the stringency for clustering. **b**, The proportion of the Y/F/WxC motif-containing secreted proteins in the RNAse supercluster or other clusters and singletons. **c**, The proposed explanation for the emergence of high-frequency conserved sequence motifs. **d**, The proposed explanation for the emergence of the hydrophobin-like effector family in *Puccinia graminis*. **e**, The network graph of cluster 25, nearly exclusive to *P. graminis*, and cluster 52, present in most fungal species in this study. Each node represents a sequence-related subcluster or singleton, and the edges indicate structural similarity between the subclusters or singletons. The size of the nodes varies on the basis of the number of members. The membership of the known virulence factors is indicated. **f**, Selected structures of the hydrophobin-like protein families. The top two structures are from *P. graminis*, while Pst_8713 and Color1_6327 are from *Puccinia striiformis* and *Colletotrichum orchidophilum*, respectively. In parentheses are pTM scores for the predicted structures.

**Extended Data Fig. 1:**
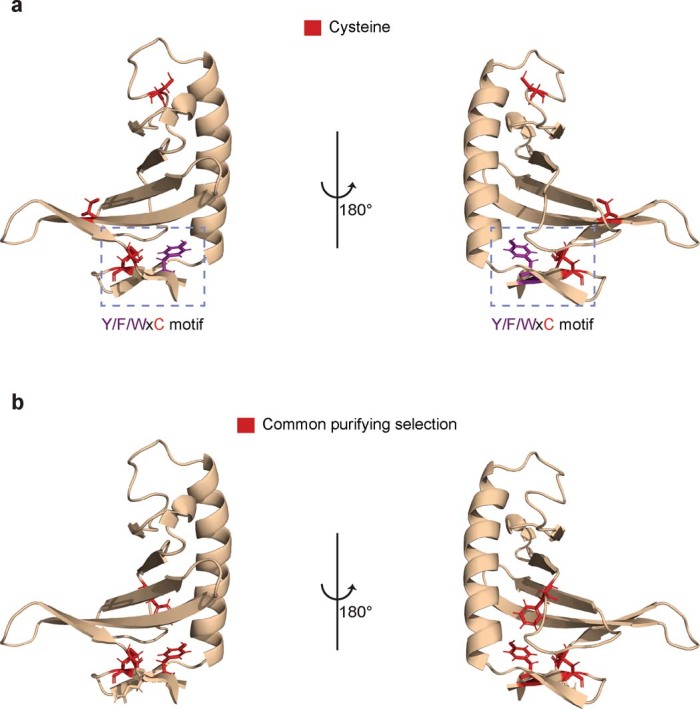
Common purifying selection on the RNAse-like effectors. **a**, A predicted structure of the RNAse-like effector cluster (cluster 5) from *Blumeria graminis* f. sp. *hordei*. All cysteine residues are highlighted in red on the structure of Blugr2_3548 with the pTM score of 0.838. The location of the Y/F/WxC motif is indicated. **b**, Four residues were found to be commonly under purifying selection in at least 5 out of 8 sequence-related subclusters in cluster 5 (Supplementary Figure 10). Their corresponding positions are colored in red on the structure of Blugr2_3548: Y2, C4, F60 and C98. The numbering of the positions is based on the mature protein sequences.

As our clustering parameters were relatively stringent, we adopted previously used parameters to recover the RNAse supercluster^7^ and retrieved 29 additional *Bgh* secreted proteins (4 clusters and 11 singletons) into the supercluster (Fig. 3a and Supplementary Table 8). A previous study curated 491 candidates for secreted effector proteins (CSEPs) and grouped them into 72 gene families and 84 singletons on the basis of sequence similarity^31^. The authors reveal that 15 different families and 7 CSEP singletons were probably RNAses. We compared the membership of the CSEPs to the RNAse supercluster and found that 60 CSEP gene families and 41 CSEP singletons belong to the supercluster (Fig. 3a and Supplementary Table 8). In other words, 70% of the CSEPs are putatively RNAses. Another previous study revealed that highly diverse putative effector groups in *B. graminis* share conserved Y/F/WxC motifs in the first 45 amino acids of the full-length proteins^32^. We found that 371 (91.4%) out of the 406 Y/F/WxC motif-containing secreted proteins belong to the RNAse-like effector supercluster (Fig. 3b and Supplementary Table 8). For *Bgh* secreted proteins other than the putative RNAses, we did not observe any enrichment of Y/F/WxC motifs (Supplementary Fig. 9). We examined site-specific purifying selection pressure on each sequence-related subcluster (Supplementary Figs 10). Despite sequence dissimilarity between the subclusters, the pair of cysteine residues forming a disulfide bond and the site for the Y/F/WxC motif were commonly under purifying selection, suggesting persistent evolutionary constraints to conserve the fold and possible importance of these residues to maintain the fold (Extended Data Fig. 1).

*Pgt* was also suggested to encode many proteins with the Y/F/WxC motif^32^, and we found that the motif was particularly enriched in cluster 25 (Supplementary Fig. 11) In this SUSS effector family, 90 (70%) out of 128 *Pgt* members contained the motif. Together, our data suggest that a high-frequency sequence motif was unlikely to emerge by unrelated proteins independently and repeatedly acquiring the motif. Instead, extreme expansion and divergent evolution of homologues that diminishes sequence similarities are more likely to explain the presence of high-frequency motifs only in some pathogenic species (Fig. 3c).

### Some SUSS effectors evolve from conserved secreted proteins

The largest SUSS effector family in *Pgt* (cluster 25) could not be characterized at the sequence level but displayed the hydrophobin-like fold nearly unique to the secretome (Fig. 2a and Supplementary Table 7). Investigating the structural similarity search results, we uncovered that cluster 25 is related to cluster 52, in which most fungal species used in this study had members, and most cluster members were annotated as fungal hydrophobins at the sequence level (Fig. 3e). Nonetheless, the extreme sequence divergence that diminished sequence similarity between the members was much more frequent in cluster 25, as most of the nodes were connected only by structural similarity. Three virulence factors, PstCTE1, PstSCR1 and Pst_8713, from *Puccinia striiformis* f. sp. *tritici* showed no detectable sequence similarity by BLAST. Nonetheless, they displayed structural similarity and belonged to the hydrophobin-like cluster (Fig. 3f). Interestingly, while PstSCR1 was shown to be an apoplastic effector^33^, PstCTE1 was suggested to localize in chloroplast^34^ and Pst_8713 in cytoplasm and the nucleus^35^, potentially reflecting functional divergence.

**Extended Data Fig. 2:**
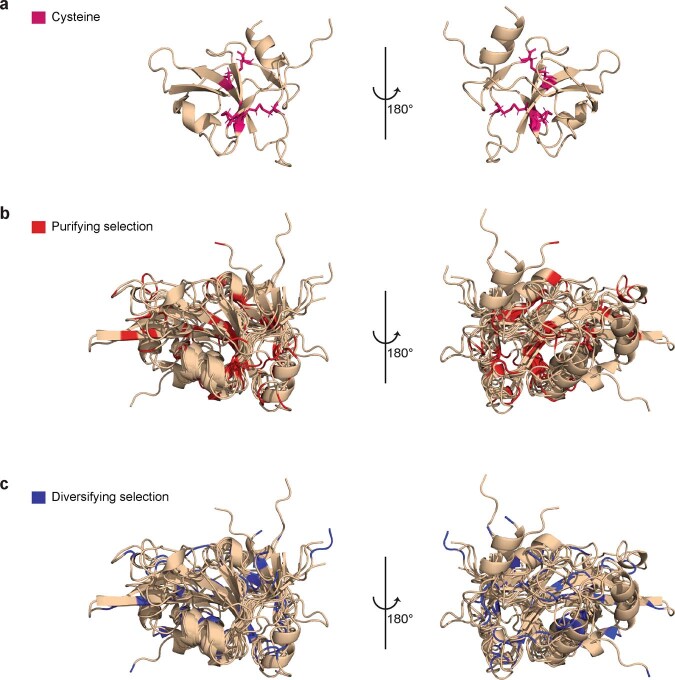
Common selection pressure on the hydrophobin-like effectors. **a**, The predicted structure of Pgt_Ug99_A1_8112 in cluster 25 with highlighted cysteine residues. **b** and **c**, The structural alignment of the representative predicted structures of hydrophobin-like effectors. A single structure was obtained from each of the nine sequence-related subclusters with five or more members. The site-level purifying and diversifying selection detected on each sequence-related subcluster was mapped to the structures (Supplementary Figure 13). The pose of Pgt_Ug99_A1_8112 remains consistent in all panels.

We examined selection pressure that might have driven the divergence of the hydrophobin-like effectors. In some sequence-related subclusters, purifying selection tended to be relaxed along the terminal branches relative to the rest of the phylogeny (Supplementary Fig. 12). However, as observed for the RNAse-like effectors, this tendency could not be generalized to all members. In almost all sequence-related subclusters, two pairs of cysteine residues, forming disulfide bonds, were under purifying selection pressure (Supplementary Fig. 13). They also constituted the most conserved structural regions when the SUSS effector structures were superposed, possibly suggesting continued evolutionary constraints to conserve the folds (Extended Data Fig. 2). Together, our data suggest that SUSS effector groups that may seem novel could have originated from conserved secreted fungal proteins (Fig. 3d). Potentially, rapid sequence divergence and subsequent acquisition of new virulent functions may be accelerating the radiation and emergence of many sequence-related subclusters, the entire connectivity of which can be discovered only by structural comparisons.

### Subcluster expansion creates genomic SUSS effector clusters

**Fig. 4:**
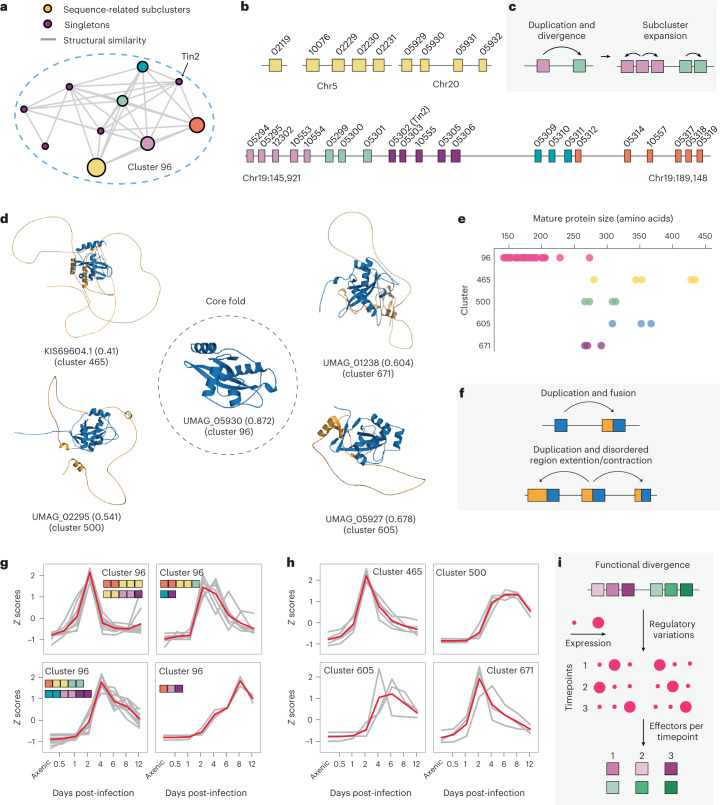
The evolution of Tin2-like effectors in *U. maydis*. **a**, The network graph of cluster 96, exclusive to *U. maydis*. Each node represents a sequence-related subcluster or singleton, and the edges indicate structural similarity between the subclusters or singletons. The size of the nodes varies on the basis of the number of members. **b**, The genomic segments that include the members of cluster 96. The colours, except purple, indicate the memberships given in **a**. The purple boxes represent singletons determined by sequence similarity searches. The two secreted proteins, UMAG_10556 and UMAG_05308 not depicted in the region of chromosome 19, do not belong to cluster 96. **c**, The proposed mechanism of the Tin2-like effector expansion. **d**, The selected structures from *U. maydis* that contain the Tin2 fold. The core Tin2 fold is coloured in blue, and the disordered stretch in orange. The pTM scores are indicated in the parentheses, and the relatively lower pTM scores are attributed to the disordered stretches that do not adopt single rigid structures. **e**, The length distributions of the mature Tin2 fold-containing cluster members. **f**, The proposed mechanism of the fusion between disordered regions and the Tin2 fold. **g**, The expression profile of cluster 96 members. The members were grouped on the basis of their similar expression patterns determined by hierarchical clustering. The membership of the sequences is indicated with coloured boxes, which correspond to the sequence-related subclusters given in **a** and **b**. The gene expressions were normalized and indicated as the *Z* scores. **h**, The expression profiles of Tin2 fold-containing disordered fusion proteins. The gene expressions were normalized and indicated as the *Z* scores. **i**, The explanation for regulatory convergence of SUSS effectors.

**Extended Data Fig. 3:**
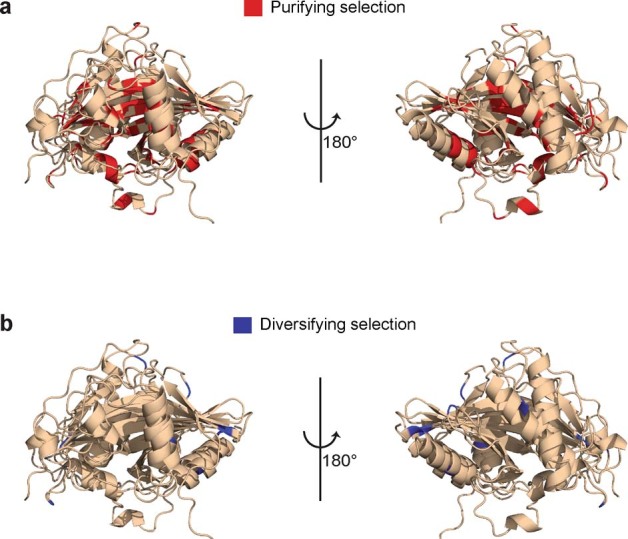
Common selection pressure on the Tin2-like effectors. **a** and **b**, The structural alignment of the representative predicted structures of Tin2-like effectors in cluster 96. A single structure was obtained from each of the four sequence-related subclusters with five or more members. The site-level purifying and diversifying selection detected on each sequence-related subcluster was mapped to the structures (Supplementary Figure 14).

Previous studies analysed a 40 kb genomic segment in chromosome 19 in *U. maydis* that contains 24 secreted effectors corresponding to five gene families and multiple singletons^36,37^. The deletion of this segment abolished the characteristic tumour formation of *U. maydis*, and Tin2 was identified as an important virulence factor that possibly alters the anthocyanin pathway in the plant hosts and reduces plant immune capabilities^37–39^. Our structure prediction and clustering suggest that the seemingly unrelated secreted proteins in this genomic segment, in fact, share structural similarity (Fig. 4a,b). Furthermore, these Tin2-like effectors form the largest, species-specific SUSS effector family in *U. maydis* with the fold found only in the secretome (Fig. 2a and Supplementary Table 7). Brefort et al. reported that there were no paralogues on other chromosomes^37^. However, structural similarity searches revealed additional Tin2-like effectors in chromosomes 5 and 20 (Fig. 4b). A plausible explanation for such SUSS effector organization is frequent subcluster expansions after sequence divergence^40^ (Fig. 4c). That is, after duplication of an ancestral Tin2-like effector occurs, one paralogue rapidly diverges, losing sequence similarity. Subsequent tandem duplications can then expand subclusters composed of paralogues in proximity that maintain detectable sequence similarity. Despite rapid divergence, positions under purifying selection were frequently detected in each sequence-related subcluster of the Tin2-like effectors, while positions under diversifying selection were barely present (Supplementary Fig. 14). When the representative structures from each sequence-related subcluster were superposed, the sites under purifying selection commonly appeared within and around the core β-strands, possibly suggesting that the sequence evolution may be constrained to maintain the structural core (Extended Data Fig. 3). Collectively, the analysis of the Tin2-like effectors suggests that a genomic array of seemingly unrelated proteins with sequence dissimilarity could originate from a single ancestral protein.

### SUSS effectors diversify through domain fusion

Structural similarity searches of Tin2-like effectors indicated the presence of additional *U. maydis*-specific clusters in which the members may adopt similar folds. Upon visualizing the structures, we found that the members in these small clusters have the core Tin2-like fold surrounded by long disordered stretches (Fig. 4d). In accordance, sequence-based disordered region prediction with IUPred2A^41^ supported that the N-terminal regions are intrinsically disordered with an abundance of glycine and proline preventing secondary structure formation (Supplementary Fig. 15). As the disordered regions could be misannotated, we relied on public transcriptomic data to confirm the gene models^42^. Even though the mature protein size varied within and between the clusters (Fig. 4e), the single-exon gene models of the fusion proteins were supported either by de novo transcriptome assembly or transcriptome mapping (Supplementary Fig. 16). Moreover, the expression of the fusion proteins was altered throughout the infection cycle with some displaying a high expression level (Supplementary Table 9), suggestive of their functional roles. Together, this supported that the ancestral Tin2 fold was fused into a disordered stretch (Fig. 4f), and its extension and contraction was followed after subsequent duplication events for possible diversification.

### Diversified SUSS effectors may converge on regulation

As high-quality transcriptomic data were available for *U. maydis*, we examined the expression profiles of the Tin2-like effectors^42^. Hierarchical clustering of the core Tin2-like cluster (cluster 96) revealed four distinct expression patterns with members from different sequence-related subclusters (Fig. 4b,g and Supplementary Fig. 17). This suggested that the members in sequence-related subclusters underwent distinct regulatory mutations (Fig. 4i), and the diverged Tin2-like effectors eventually reconverged in effector regulation, potentially diversifying functional pools of effectors. On the contrary, the Tin2 fusion proteins, which have generally maintained sequence similarity among the members within the same cluster, tended to display similar expression profiles by clusters (Fig. 4h), possibly complementing the core Tin2-like effectors’ roles.

### SUSS proteins may not be unique to phytopathogens

**Fig. 5:**
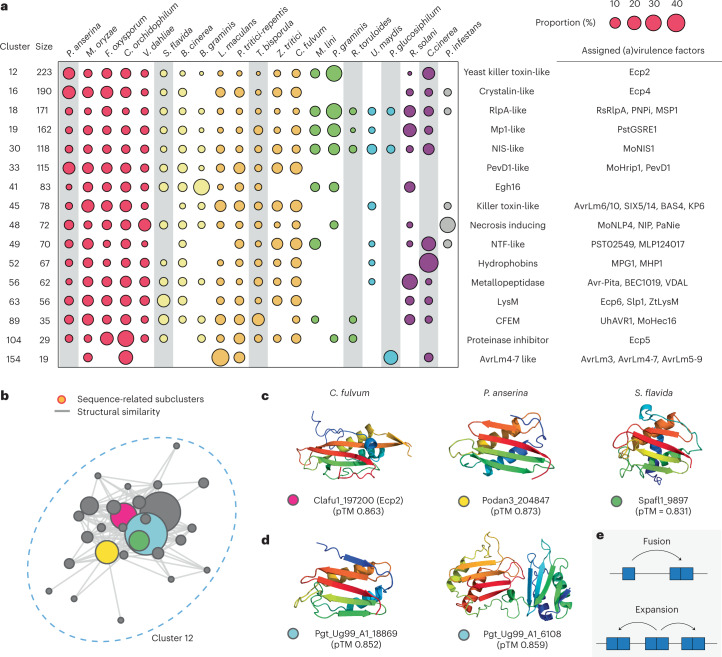
Divergent evolution of the commonly shared clusters. **a**, The putative effector families shared between phytopathogens and non-phytopathogens. The relative compositions of individual species in each cluster are indicated with circles of varying sizes. Only a subset of known virulence factors are indicated (Supplementary Table 5). Non-phytopathogenic species are highlighted with grey boxes. **b**, The network graph of cluster 12. Each node represents a sequence-related subcluster or singleton, and the edges indicate structural similarity between the subclusters or singletons. The size of the nodes varies on the basis of the number of members. The light-blue subcluster contains secreted proteins only from *P.graminis* and *M. lini*. **c**, The selected predicted structures from different sequence-related subclusters. The membership of the secreted proteins is indicated with coloured dots that correspond to the subclusters given in **b**. **d**, The selected predicted structures from *P. graminis*. These proteins belong to the light-blue subcluster specific to *P. graminis* and *M. lini*. **e**, The explanation for the emergence of novel dual-domain proteins in *P. graminis*.

We next examined clusters that are not specific to phytopathogens (Fig. 5a and Supplementary Table 5). Despite the presence of known virulence factors, many clusters included members from non-phytopathogens, on the basis of sensitive sequence similarity searches and structural comparisons. A large, shared cluster without a definitive role assigned to secreted proteins is cluster 12 (Fig. 5b). Extracellular Ecp2 from *C. fulvum* with necrosis-inducing factor domain (PF14856) belongs to this cluster. Nonetheless, about 30% of the cluster members originated from non-pathogenic species. The analysis of the network indicated that sequence-unrelated structural similarity was not necessarily a unique feature of phytopathogens, and identifying related proteins required structural comparisons for non-pathogenic species (Fig. 5c). Interestingly, in the sequence-related subcluster that only includes members from *Pgt* and *M. lini*, two yeast killer toxin-like domains were fused in a single protein (Fig. 5d,e). This fusion protein was supported by transcriptomic data^43^ and appeared to be expanded in *Pgt* (Supplementary Table 4). Collectively, such distinct evolution may reflect different evolutionary pressures on these SUSS groups from which virulence factors could evolve. That is, similar to the RNAse-like and hydrophobin-like effector families in *Bgh* and *Pgt*, virulence factors may originate by divergent evolution of inherited secreted proteins, as an outcome of adaptation.

## Discussion

Primary sequences of many fungal effectors cannot provide sufficient information about their evolution or adequately depict the diversity of these rapidly evolving proteins. Tools such as effectorP^44^ can guide the prediction of effectors through the classification of secreted proteins on the basis of known effectors’ features; yet, they do not illuminate evolutionary or functional information. Computational structural genomics offers more intuitive information about the effectors by revealing their structural similarity to the existing and novel effector families. A comparative study across pathogens further extends the evolutionary context and reveals additional clues about effectors that the studies on single species may not capture. Through this study, we demonstrate the advantages of comparative computational structural genomics and how this method can reveal novel evolutionary insights about effectors masked by their sequence dissimilarities.

**Fig. 6:**
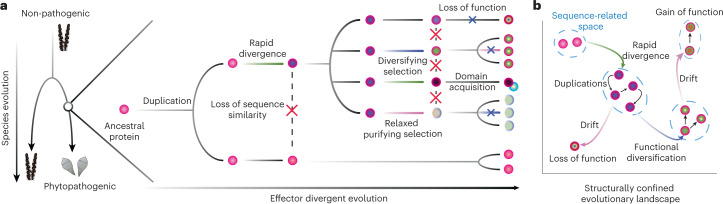
The divergent evolution of effectors. **a**, The proposed evolution of a phytopathogen from an ancestral non-phytopathogenic species and the emergence of effector families from ancestral proteins. A protein that was present in the ancestral species undergoes a duplication event. A paralogue rapidly diverges and loses sequence similarity to the other paralogue. Such processes occur repeatedly, leading to contemporary protein groups that are not related by their sequences. **b**, The evolution of SUSS effectors. The proteins exist in a structurally confined space. Through rapid divergence and duplications, SUSS effector groups emerge, occupying a novel sequence-confined space that may have distinct functions or roles.

Our study primarily underscores divergent evolution of fungal secreted proteins (Fig. 6). In this model, a protein present in a common ancestor of non-phytopathogenic and phytopathogenic species can evolve to form SUSS effector groups (Fig. 6a). After a duplication event of the ancestral protein, a paralogue rapidly diverges in primary sequences and loses sequence similarity to the other paralogue. The paralogue may keep increasing its copies, some of which would be continuously selected through diversifying selection or quickly accumulate new mutations after purifying selection becomes relaxed. During this process, evolutionary constraints to conserve the folds probably remain persistent on the structural core. In particular, conserved disulfide bonds may effectively restrict the effector divergence process into the structurally confined landscape. Eventually, multiple effector groups emerge with no evolutionary connections on the basis of primary sequences. The connections of these groups can be elucidated by the structural comparison.

SUSS effectors probably have distinct virulent functions and roles. For instance, different localizations of hydrophobin-like effectors in *Pgt* may reflect biochemical or biological specialization^33–35^. TIN2, TIN4 and TIN5 of *U. maydis* contribute to virulence and tumour formation to a varying degree^37^. MAX effectors display unique surface properties, potentially suggesting distinct host targets^3^. In this sense, the emergence of some SUSS effectors could be a natural outcome of functional divergence. The rapid sequence divergence that diminishes sequence similarities between homologues may result from accelerating neo-functionalization. Alternatively, acquiring novel and strong virulent functions may be only accomplished by accessing other sequence-confined evolutionary realms within the structurally confined evolutionary landscape (Fig. 6b). Rapid sequence divergence may be, therefore, a necessary process for functional diversification. Nonetheless, not all SUSS effectors would diversify for new functions (Fig. 6). Our data support that purifying selection was relaxed along the most recent duplications of some SUSS effectors, allowing accumulation of new mutations. Other SUSS effectors may be experiencing extensive drift without definitive roles and functions. Although a subset of these effectors may acquire new functions in the long run, others may eventually be lost (Fig. 6b).

The protein structure space is more confined than the protein sequence space^45^. The constraints may restrict unrelated sequences to adopt similar folds. Proteins may also independently evolve to form similar structures to solve similar biological problems. That is, not all SUSS effectors will be an outcome of divergent evolution, and whether divergent evolution or convergent evolution favours the emergence of certain effector clusters would require more rigorous evaluation in primary sequences, protein structures and genomic contexts. However, recurring examples of linking pathogen effectors with non-pathogenic homologues make divergent evolution more plausible to explain the root of effectors. Furthermore, this model is in accordance with our observations on (1) similar compositions of secretomes between evolutionarily closely related species even after structure-based clustering, (2) the presence of virulence factor-containing clusters shared between pathogens and non-pathogens, (3) the existence of highly expanded effector groups, such as hydrophobins in *Pgt* and RNAses in *Bgh*, that potentially originated from conserved secreted proteins and (4) numerous, independent emergence of fungal parasitism. Under the divergent evolution hypothesis, any secreted protein may evolve virulence functions and form SUSS effector families. Finding other distinct classes of such protein families would not be unexpected. This also could imply that there are numerous different solutions to evolve pathogenicity. However, the divergent evolution model poses challenges in that a single, definitive role cannot be assigned to each effector family. Rigorous molecular biology, guided by structure-based evolutionary studies, will remain essential to deepen our understanding of functional divergence and unique utilization of effectors.

### Finer species sampling for better evolutionary resolution

We believe that ancestral origins of many species-specific effector families can be revealed through finer sampling of fungal species. Although MAX effectors were nearly exclusive to *M. oryzae* in our study, *V. inaequalis* in Ascomycota was suggested to encode many MAX effector-like proteins^21^. Similarly, *Sporisorium reilianum* related to *U. maydis* encodes Tin2-like effectors found exclusively in *U. maydis* in our study^46^. Yeast-like *Pseudozyma hubeiensis* believed to be non-pathogenic seemed to also share homologues (for example, XP_012191528.1) (refs. ^47,48^). Therefore, resolving the ancestral origins of all effector folds will require a larger-scale comparative study with finer samples.

### Singletons: missed prediction or true singletons

Although our study revealed important features of effector evolution, it may not yet provide a comprehensive perspective of pathogen effectoromes. Some putative virulence factors might have not been properly annotated or correctly predicted to be secreted, leading to the underestimation of effector family sizes. Seven thousand two hundred seven (27%) secreted proteins remained as singletons and were therefore not discussed. Most of these singletons are missing predicted structures. Some of the singletons may be true singletons without any evolutionarily related proteins in other species or within the species; others may not be. For instance, a recent structural genomics study highlighted FOLD effectors in *F. oxysporum* f. sp. *lycopersici*, represented with Avr1 (SIX4), Avr3 (SIX1), SIX6 and SIX13 (ref. ^8^). Some of the proteins were not modelled with pTM scores >0.5, and many putative FOLD effectors remained as singletons in our study. This possibly suggests that some structural folds may be harder to predict with AF2, and many potentially expanded novel folds may be hidden in the singletons. Benchmarking AF2’s prediction with experimentally determined structures revealed that the effector folds could still be predicted with relatively low estimated precision^8^. In such cases, lowering the criteria for predicted structure selection and structure-based clustering could be helpful, as shown in our previous study^7^. Alternatively, language model-based structure prediction software, such as OmegaFold and ESMFold, may better predict the structures of the singletons than AF2 (refs. ^49,50^).

### Disordered proteins: understudied players of pathogenesis

We found that the core Tin2 fold of *U. maydis* may be diversifying by fusing with disordered stretches and subsequently contracting and extending them. This could be a strategy for a more rapid functional specialization than accumulating point mutations. As shown for other effectors, some portions of these disordered stretches could be removed by Kex2 before secretion^8,51,52^. Although many of the Tin2 fusion proteins contain putative Kex2 cleavage motifs (KR, RR and LxxR)^52^, the location of the motifs does not precisely distinguish the disordered region and the conserved core fold. The remaining intrinsically disordered regions may provide advantages to effectors, for instance, by aiding effector translocation^53^. The additional importance may lie in the interaction between effectors and host immunity. The flexibility of the disordered regions and the absence of a single rigid conformation would not provide sufficient opportunities for the plant immune receptors to evolve specificity. As sequence evolution occurs much faster on the long disordered region^54^, evading recognition could be accomplished more easily. Such features may drive the intrinsically disordered stretches to function as a shield of the core effector folds, reducing the frequency of the encounter between the core folds and immune receptors, while hindering the evolution of recognition specificity. Molecular biology will be an important avenue to elucidate how disordered effectors may function to compromise plant immunity.

### Predicted structures as a resource for future studies

The predicted structures generated in this study can serve as resources for larger comparative studies. The expansion to other fungal pathogens that infect humans, mammals and insects, as well as finely sampled non-pathogenic fungal species could illuminate the distinct evolution of diverse lineages across the fungal kingdom. Structure-guided evolutionary studies on plant-infecting bacteria, nematodes and insects may also elucidate further insights into the plant–pathogen interactions.

## Methods

### Secretome prediction

The protein sequences of the species used in this study were downloaded from the Joint Genome Institute (JGI) and Ensembl Fungi (Supplementary Table 1)^24,55–74^. We used the neural network of SignalP v3.0 to identify secreted proteins^75^. The candidates were excluded if their predicted signal peptides overlapped with PFAM domains annotated with InterProscan v5.30-69.0 over ten or more amino acids^76^, or if their mature proteins contained any transmembrane helices detected with TMHMM v2.0 (ref. ^77^). Only the mature proteins 15–860 amino acids in length were selected for structure modelling.

### Structure prediction

The structures of 26,653 sequences were predicted by AF2 (ref. ^20^). For secreted proteins, the signal peptides were removed before modelling. The full databases were used for multiple sequence alignment (MSA) construction, with an additional 1,689 fungal protein sequences downloaded from the Joint Genome Institute appended to the UniRef90 database. All templates downloaded on 20 July 2021 were allowed for structural modelling. When the generated MSA was too large to process in our machine (>1 GB), we used HHfilter v3.3.0 to reduce the redundancy^78^. For each protein, five models were generated with model_1, 3, 4 and 5, as well as model_2_ptm to obtain the pTM score. We selected the best model (ranked_0.pdb) determined by the average pLDDT score. For the non-secreted proteins, unless the predicted structures are already available^79^, we followed the same pipeline to model the structures. The only difference was that the homologues were collected only from the UniRef90 and MGnify databases, also allowing the homologous templates.

### Functional and structural annotations

The functional annotation was performed against Gene 3D v4.3.0, PFAM v33.1 and Superfamily v1.75 with InterProscan v5.52–86.0 (refs. ^80–82^). We used Rupee for structural similarity search against SCOPe v2.07, CATH v4.3.0 and PDB chain databases downloaded on 2 September 2021 (TOP_ALIGNED, FULL_LENGTH)^83,84^.

### Protein similarity searches

Sequence similarity searches were performed with BLASTP v2.7.1+, HHblits v3.3.0 and HHsearch v3.3.0 for 26,653 secreted proteins^78,85^. HHblits and HHsearch require a sequence profile generated with an MSA. The profile was constructed by concatenating all MSAs produced by AF2 and filtering the concatenated MSA with HHfilter v3.3.0 (-id 90 -cov 50 -maxseq 20000). All sequence similarity search outputs were filtered on the basis of *E*-value (expect value) ≤1 × 10^−10^ and bidirectional coverage ≥65% before clustering. Structural similarity search was performed with TM-align^86^. Two structures were considered similar if they were predicted with pTM scores >0.5, and their structural similarity was measured with TM score >0.5 normalized for both structures. The parameters were set more stringent than the criteria used in our previous work to reduce false clustering^7^. To identify the RNAse supercluster in *B. graminis*, the previously used parameters were adopted: *E*-value <1 × 10^−4^ and bidirectional coverage >50% for sequence similarity searches, and TM scores >0.5 for both structures or TM scores >0.6 and >0.4 for each structure for structural similarity searches.

### Clustering and network analysis

Protein clustering was performed sequentially with the similarity search outputs from BLASTP, HHblits, HHsearch and TM-align. Unlike our previous study that relied on a connected network^7^, we applied the Markov clustering algorithm to reduce false clustering with the mcl package v14-137 (ref. ^87^). We first generated with mcxload a network of protein sequences on the basis of −log_10_ (*E*-value) as weights from the BLASTP similarity search results, while capping the weight at 200 (–stream-mirror–stream-neg-log_10_ -stream-tf ‘ceil(200)’). We then defined clusters with mcl with an inflation factor of 2 (-I 2.0). Once the protein sequences are assigned into clusters, the pairwise sequence similarity search outputs from HHblits were redefined to indicate the connectivity between the clusters. The average weights between the members in two clusters were used as the weight between the clusters, similarly to the average-linkage clustering. These processes were repeated for HHsearch and TM-align similarity search results as well. For structure-based clustering, as the TM scores range from 0 to 1, these scores were used directly without converting them to a log scale. The final clusters were defined as and used interchangeably with families. We used networkx v2.2 to visualize the network graphs^88^.

### Motif analyses

The Y/F/WxC motif was identified by scanning 3-mer in a sliding window in the first 45 amino acids of the secreted proteins^32^. Kex2 cleavage motifs were predicted by searching for KR, RR and LxxR motifs in the N-terminal disordered stretches of mature Tin2 fusion proteins in clusters 465, 600, 605 and 671 (ref. ^52^).

### Selection pressure analyses

In the final sequence-related subcluster, the mature protein with the highest pTM score was selected as a reference. The sequence profile of the reference and hmmalign v3.1b2 (ref. ^89^) were used to align the mature sequences of the subcluster members, and the columns in which more than 50% positions were gaps were removed. This trimmed MSA was used to infer a phylogenetic tree with FastTree v2.1.11 (ref. ^90^) (-slow) and to generate a codon alignment. We used HyPhy v2.5.41 (ref. ^91^) to examine selection pressures. Site-level positive and negative selection was detected with FEL v2.1 (ref. ^92^) and MEME v3.0 (ref. ^93^) by testing all branches. Branch-level diversifying selection pressure was inferred with aBSREL v2.3 (ref. ^94^) by testing all branches. Branch-level relaxed or intensified purifying selection was identified with RELAX v3.1.1 (ref. ^95^). The branches of the terminal leaves were tested against the rest of the branches to examine whether purifying selection was altered after the most recent duplication events. Multiple structures were aligned by mTM-align to examine if the sites under selection pressure overlap in the structures^96^.

### Reporting summary

Further information on research design is available in the Nature Portfolio Reporting Summary linked to this article.

## Supplementary information

Supplementary Information Supplementary Figs. 1–17.

Reporting Summary

Supplementary Table Supplementary Tables 1–9.

## Data Availability

All the datasets and scripts used in this study can be downloaded from Zenodo (10.5281/zenodo.6480453)^97^.
