# Supplementary material for: Prediction of effector protein structures from fungal phytopathogens enables evolutionary analyses
Source: Nat Microbiol. 2023 Jan 5;8(1):174–87. doi: 10.1038/s41564-022-01287-6 (PMC9816061; doi:10.1038/s41564-022-01287-6)
Supplement: Supplementary file 1 — Supplementary Figs. 1–17. [file 41564_2022_1287_MOESM1_ESM.pdf]

nature microbiology

Resource

https://doi.org/10.1038/s41564-022-01287-6

Prediction of effector protein structures from fungal phytopathogens enables evolutionary analyses

In the format provided by the authors and unedited

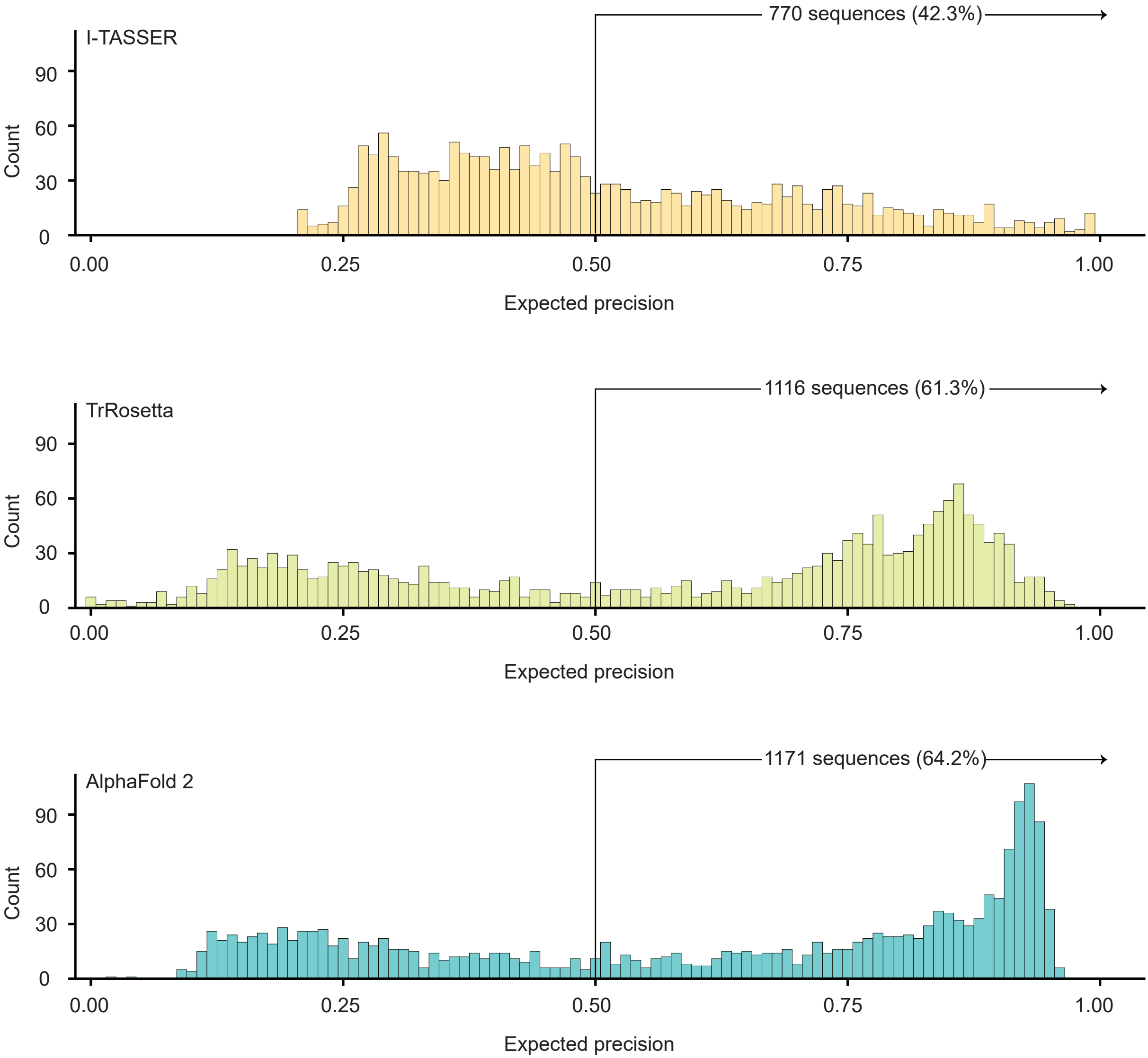

Supplementary Figure 1. The comparison of estimated precision from I-TASSER, TrRosetta and AlphaFold 2 for the predicted structures of *Magnaporthe oryzae* proteins.

The distribution of expected precision from the three structure prediction tools for the same set of 1,822 secreted proteins from *Magnaporthe oryzae*. The number of sequences, the structures of which were predicted with expected precision > 0.5, are indicated.

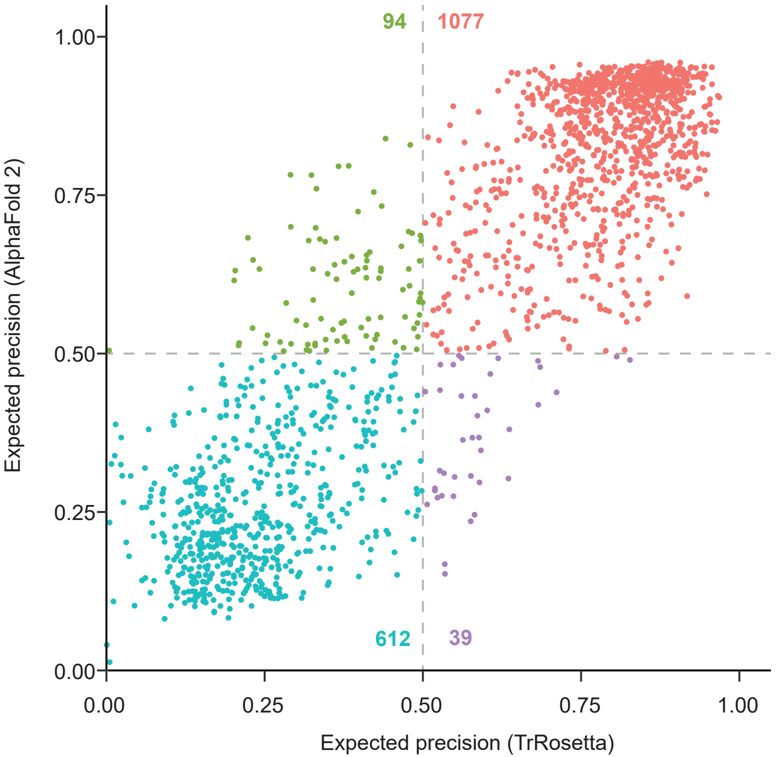

Supplementary Figure 2. The comparison of expected precision for AlphaFold 2 and TrRosetta structures for *Magnaporthe oryzae*'s secreted proteins.

The distribution of expected precision from AlphaFold 2 and TrRosetta for the same set of 1,822 secreted proteins from *Magnaporthe oryzae* is displayed in the Cartesian coordinate. The number of sequences that belong to each coordinate is indicated.

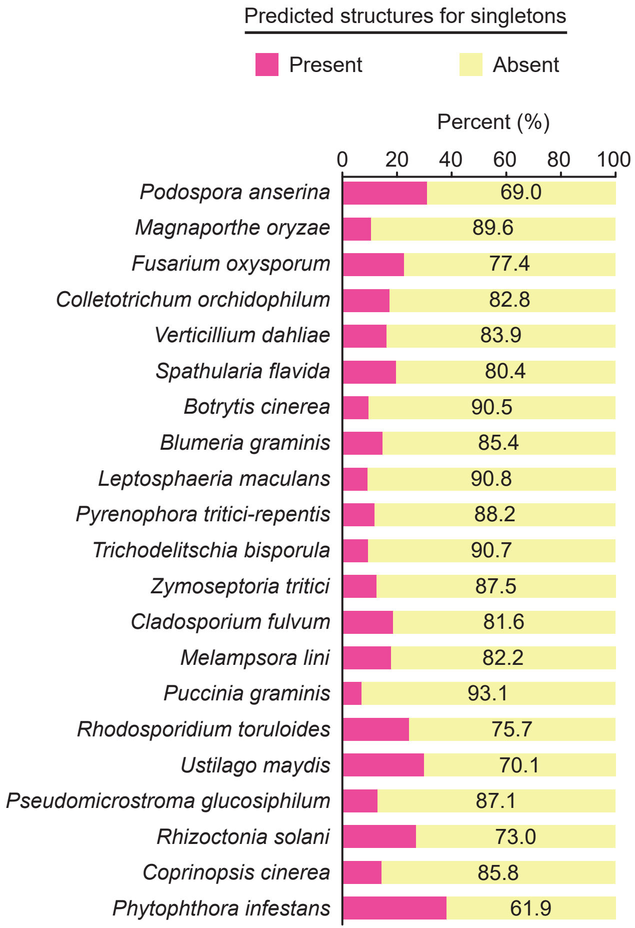

Supplementary Figure 3. The structure prediction statistics for singletons.

The proportions of singletons in each species that have or do not have predicted structures are indicated. These singletons did not have any sequence or structure-related proteins in both individual and whole-secretome clustering.

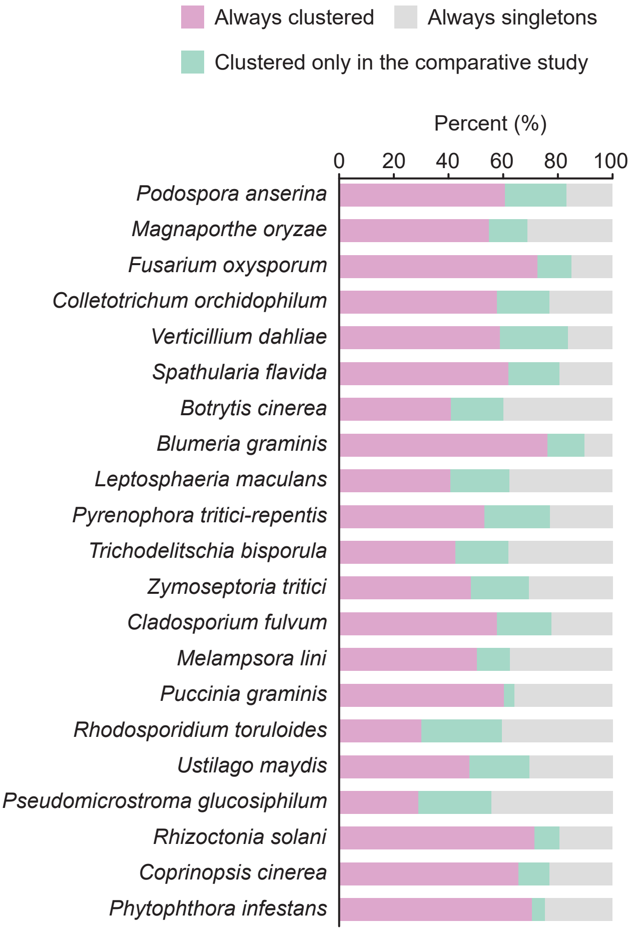

Supplementary Figure 4. The statistics of secretome clustering.

The secretome of individual species or entire 21 species in this study were clustered based on sequence and structural similarities. The secreted proteins were categorized into 'Always clustered' if the proteins appear in a cluster in both individual and whole-secretome clustering outputs, 'Clustered only in the comparative study' if the proteins appear in a cluster only in the whole-secretome clustering output, and 'Always singletons' if the proteins appear as singletons.

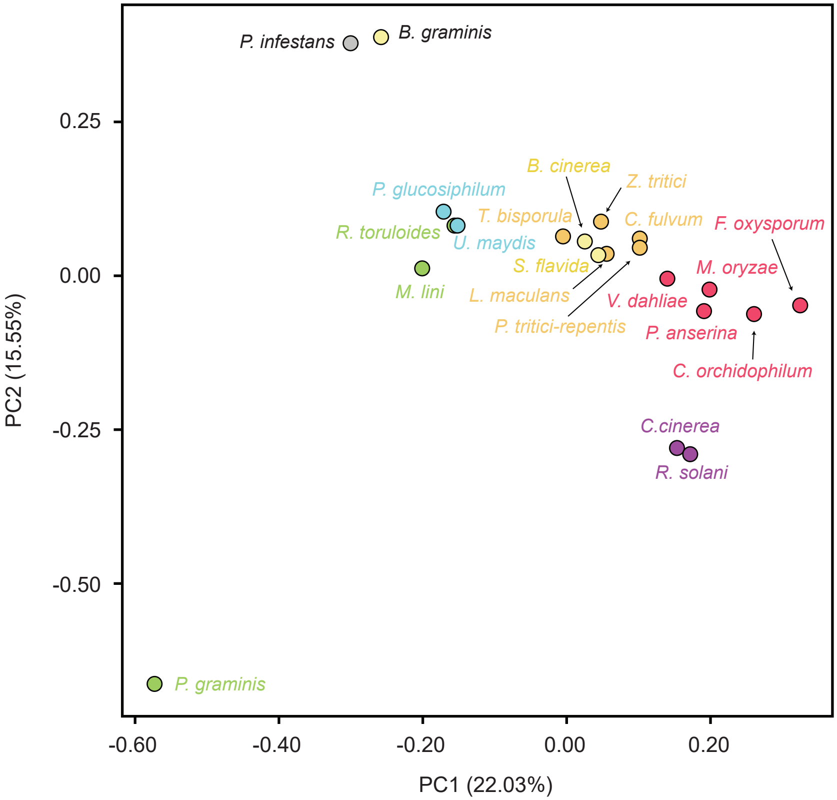

Supplementary Figure 5. The principal component analysis on BLAST-based clustering outputs.

The secretome of the 21 species was clustered only based on sequence similarity search results obtained with BLASTP. The scaled centered cluster counts were reduced into the two dimensions with the principal component analysis. The color of the circles is given based on the taxonomy (class or subdivision) following Figure 1a.

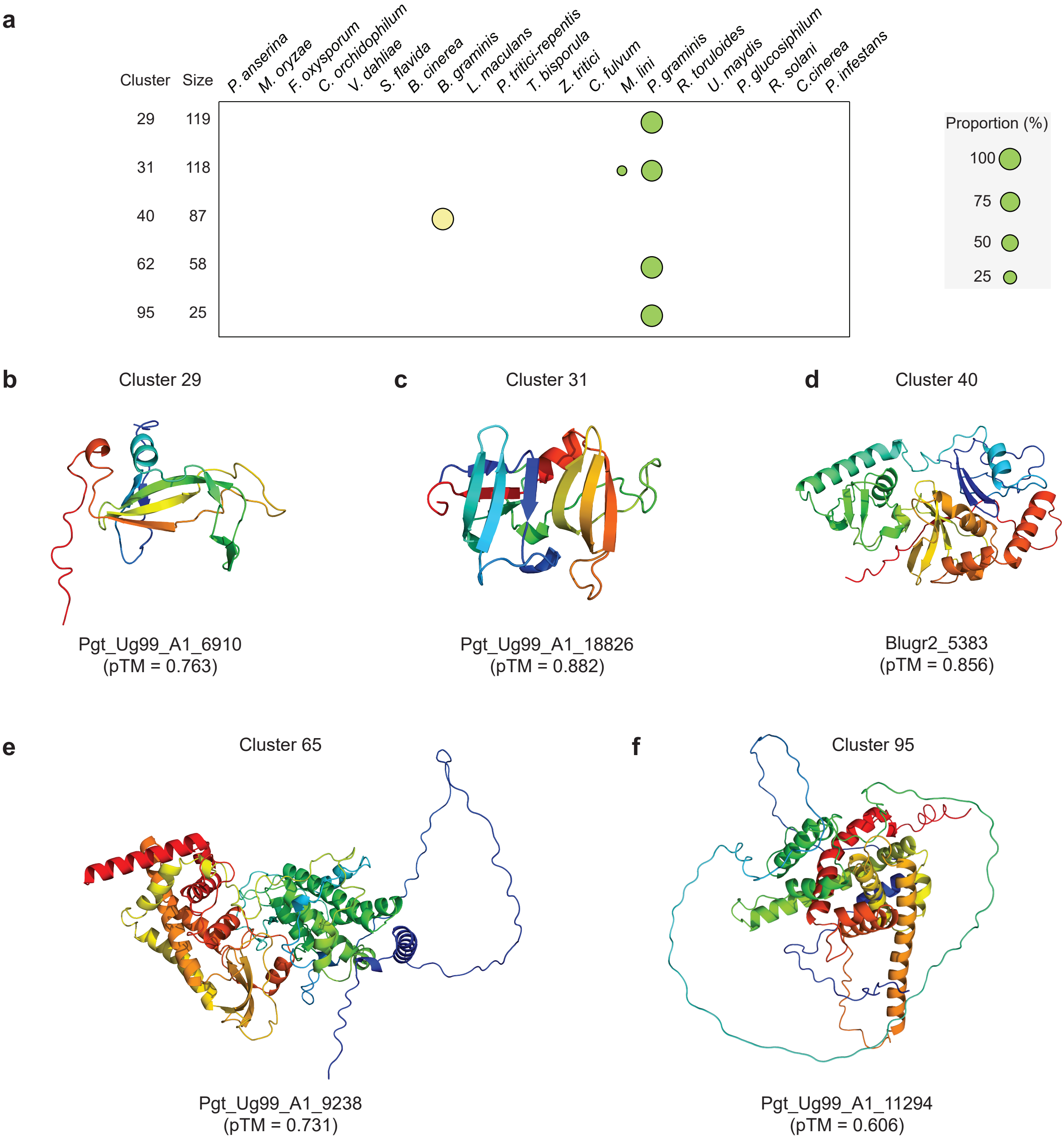

Supplementary Figure 6. Nearly or entirely species-specific clusters without known virulence factors.

a, Nearly or entirely species-specific clusters found in *Blumeria graminis* and *Puccinia graminis*. The relative composition of each species is indicated with circles with varying sizes. **b – f,** The selected predicted structures from each cluster.

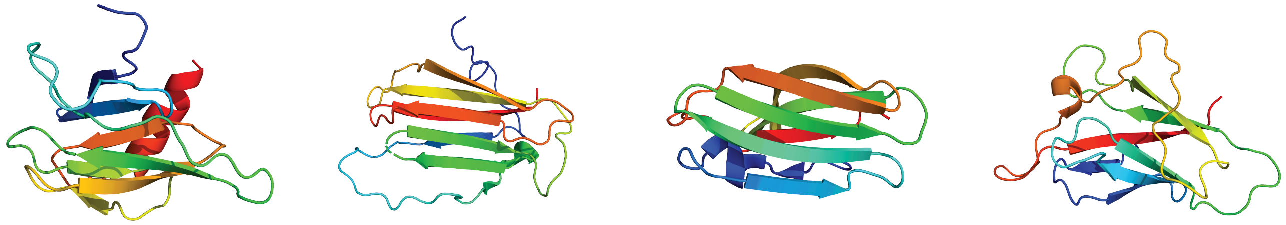

AvrSr50 (7MQQ) | Avr2 (5OD4) | ToxA (1ZLD) | AvrL567(2OPC)

Supplementary Figure 7. Selected effectors that contain β-sandwich-like folds.

Experimentally determined structures of AvrSr50 (Cluster 171), Avr2 (Cluster 68), ToxA (Cluster 68) and AvrL567 (Cluster 1283). The PDB accessions are given in the parentheses.

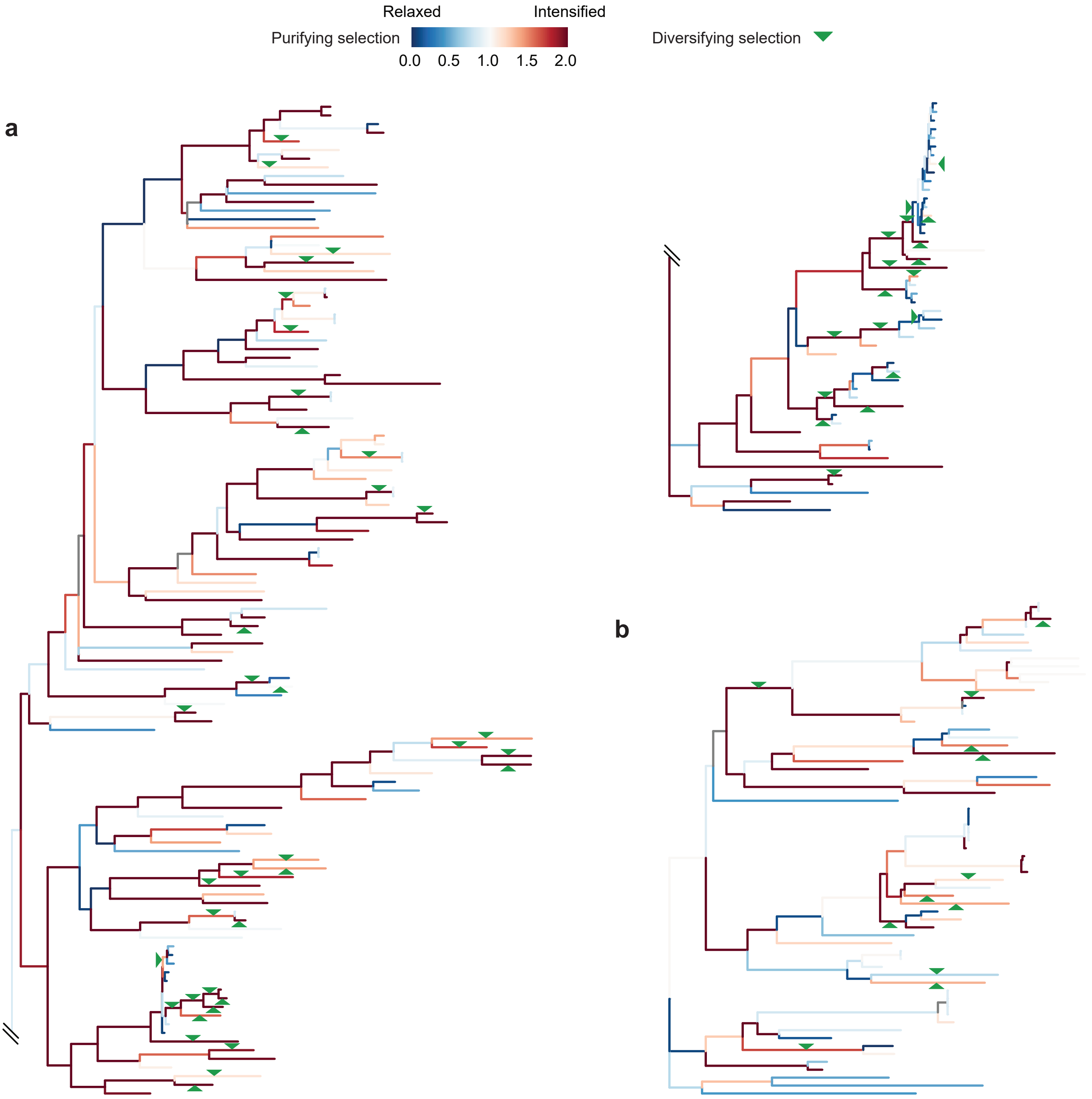

Supplementary Figure 8. Branch-level selection pressure on sequence-related subclusters of the RNAse-like effector group (Cluster 5) in *Blumeria graminis* f. sp. *hordei*.

The intensity of purifying selection on the terminal branches (test branches) were compared to the rest (reference branches) with RELAX to determine whether the purifying selection was overall relaxed or intensified after the most recent duplications that led to the terminal leaves. The significance of relaxation or intensification is captured through a likelihood ratio test computed by RELAX. For visualization, the descriptive selection intensity parameter k was normalized using geometric means, after limiting the maximum value of k to 10. The normalized k values were mapped to the branches, with the maximum normalized value capped at 2. The branch-level diversifying selection was examined with aBSREL, and the branches under diversifying selection ($p < 0.05$) are indicated with green triangles. **a**, HHsearch subcluster 10 with 163 members. The relaxation of selection was detected among the test branches relative to the reference branches ($p = 0.0000$). 55 branches were under diversifying selection. The tree was split into two parts only for visualization. **b**, HHsearch cluster 43 with 63 members. The relaxation of selection was detected among the test branches relative to the reference branches ($p = 0.0095$). 13 branches were under diversifying selection.

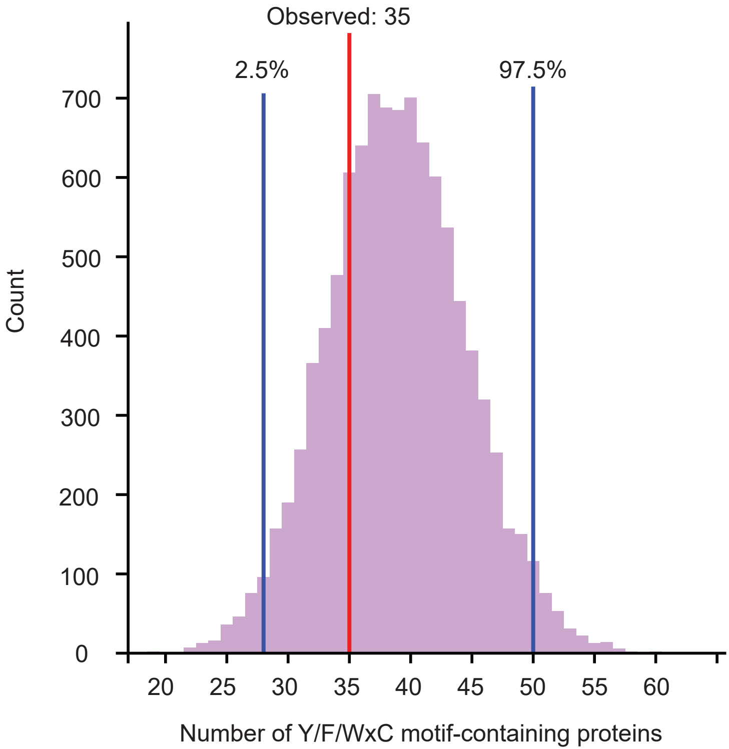

Supplementary Figure 9. The distribution of the number of Y/F/WxC motifs in the permutation test.

For non-RNAses, the first 45 amino acids were permutated and the Y/F/WxC motif was searched. This permutation test was performed 10,000 times, and the distribution of the number of Y/F/WxC motif-containing sequences is provided. 2.5% and 97.5% quantiles are indicated. The observed 33 Y/F/WxC motif-containing sequences could occur by chance.

```
Blugr2_1350    -------------------------LHIPGVSKTEKKKPKPLRTGYRCDAIDFPEDSGWG
Blugr2_2422    --GFLNAKATGNSQPLRVPIQE--K-----------------PFYITCGS---------G
Blugr2_281     ------------------------------------------DGYHECSY---------G
Blugr2_3176    -------------------------------------------ADYICDN---------E
Blugr2_3548    --------------------------------------------VYKCPS---------Q
Blugr2_3826    ------------------------------------------EYKYVCPS---------K
Blugr2_4419    ------------------------------------------AIYYNC-L---------D
Blugr2_6598    TF-------------TASHI-NEV------------------NKGFACE----------G

Blugr2_1350    NSYRIEGLRK--VVKQSCGIAK-K---R--L-K--MTEKQKLDREKLSFWIQD-------
Blugr2_2422    SSYDYSYLES--SVHEGLSRI--S---DL-------S------K--------------GQ
Blugr2_281     KVFPMSEIRH--KLEFG----------N-F------------------------------
Blugr2_3176    AEVPAKFVND--FLSHARVQARNRQSGY--SLENP-------------------------
Blugr2_3548    QYIDDFTINT--AANNLYEKGL-Q---F--N-F--GRH---------------PGQSEC-
Blugr2_3826    IKFTTARIDM--LRNDISSGS------I------------------------------T-
Blugr2_4419    AYIPKTEIDE--RVRAKYQDLVAQ---G--TRP---------------------------
Blugr2_6598    RLFMHEEYNRVE----K-------------------------------------------

Blugr2_1350    DPYP-MP--------FP-QYEK-F-----------G---F----S---------KEA-QI
Blugr2_2422    EAYP-IE--------NT-D----F-----------V---YKV----------DGK-Y-WV
Blugr2_281     ---E-DAR----DT--D-P-T--------------GPND-------E-------E-Y-FG
Blugr2_3176    ---G-DV------------YS-------------------------G----GRNP-L-YW
Blugr2_3548    GG-I-IF--------------------------------------SG--STANHDLT-FT
Blugr2_3826    EG-V-LI--------------------------------------IT--S--SGLRV-LT
Blugr2_4419    -----------------------------------------------DQ-FQAGS-TFGH
Blugr2_6598    --MELTGPVNELGYTMSYIYDNLLQDIKDRRICAY-----QDSY--------ETE-Y-QF

Blugr2_1350    MPL-PRP--------MQ-TEPG---------------SDFVVFQPSDC---KVLSVVQVS
Blugr2_2422    YPI-LHTNQVYQQGQ-----VD---------------DDFVVFNS-KG---LILGGLHL-
Blugr2_281     MRF-DM----------------MPV--PE-----VTVTYLIQII-Y--KAP-YYRFYEE-
Blugr2_3176    RAI-IVS--------QDL----ERFNG-------QHYEYRIVYDS-QF---NLHKLEAV-
Blugr2_3548    RAF-RPP--------FT-----TV------------MSYKLQVSHP--S--KQITLIEC-
Blugr2_3826    YIF-DR----------------KI-ID------GVAYDFMVTVNYR--D--KLTRVYEK-
Blugr2_4419    VSY-N------------R----ESVLNRH-----GSMEITVKFDK-SK---NIISVKAL-
Blugr2_6598    FELTNS---------------WQS--Q-LLHNGHLVHAYILVIDS-YN---RANAMIRR-
                                             6
Blugr2_1350    RDK-------------VPGN-YHSCRIA-Q-----WNKTLLDWLWPNN-
Blugr2_2422    DKT------S-----PR---TVYPCKFSETTEFMP--------------
Blugr2_281     T-TS----------------GWRPCPFL-D-------------------
Blugr2_3176    RLN-G----GGS----E---TTFHCYSY---------------------
Blugr2_3548    G-I---------TEEGY---TEKACQKQ---------------------
Blugr2_3826    E-S-------------E---HTRICELI-F-----VL-----------D
Blugr2_4419    Y-S-------------G---NNYNCQVF-H-----WE------------
Blugr2_6598    KTI-FEGQRSPK----V---TYSICEIR---------------------
```

Site-specific selection

Purifying

Pervasive diversifying

Episodic diversifying

Supplementary Figure 10. Selection pressure on the RNAse-like effector group in *Blumeria graminis* f. sp. *hordei*.

The alignment of representative sequences from 8 sequence-related subclusters in Cluster 5: Blugr2_2422 (HHsearch cluster 10; 163 members), Blugr2_281 (HHsearch cluster 43; 63 members), Blugr2_6598 (HHsearch cluster 74; 43 members), Blugr2_1350 (HHsearch cluster 115; 28 members), Blugr2_4419 (HHsearch cluster 123; 27 members), Blugr2_3548 (HHsearch cluster 327; 10 members), Blugr2_3176 (HHsearch cluster 374; 9 members), and Blugr2_3826 (HHsearch cluster 453; 7 members). These representative sequences have the highest pTM score in each subcluster. The alignment was constructed with mTM-align by superposing the predicted structures of the eight members. For each subcluster, purifying selection and pervasive diversifying selection were detected with FEL, and episodic diversifying selection was inferred with MEME from HyPhy. The sites under selection pressure were colored. If episodic diversifying selection was detected on the sites under purifying or pervasive diversifying selection, it is not shown.

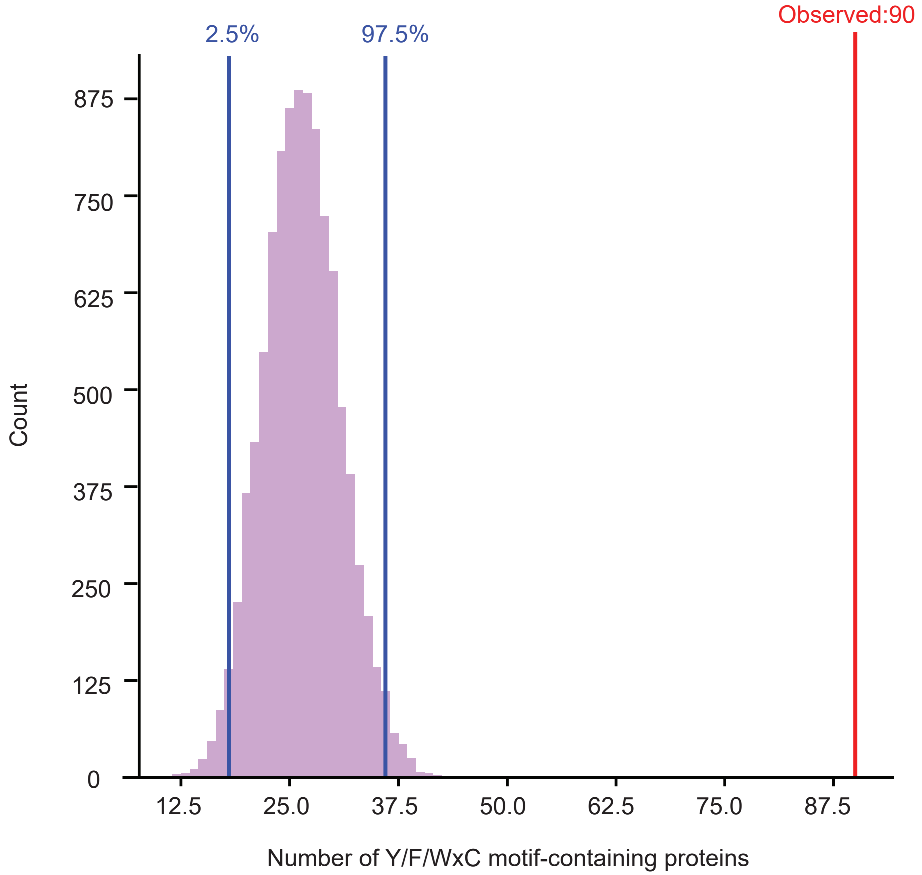

Supplemental Figure 11. The distribution of the number of Y/F/WxC motifs in the permutation test for Cluster 25 members in *Puccinia graminis*.

For Cluster 25 members from *Puccinia graminis*, the first 45 amino acids were permutated and the Y/F/WxC motif was searched. This permutation test was performed 10,000 times, and the distribution of the number of Y/F/WxC motif-containing sequences is provided. 2.5% and 97.5% quantiles are indicated. The observed 90 Y/F/WxC motif-containing sequences unlikely occur by chance.

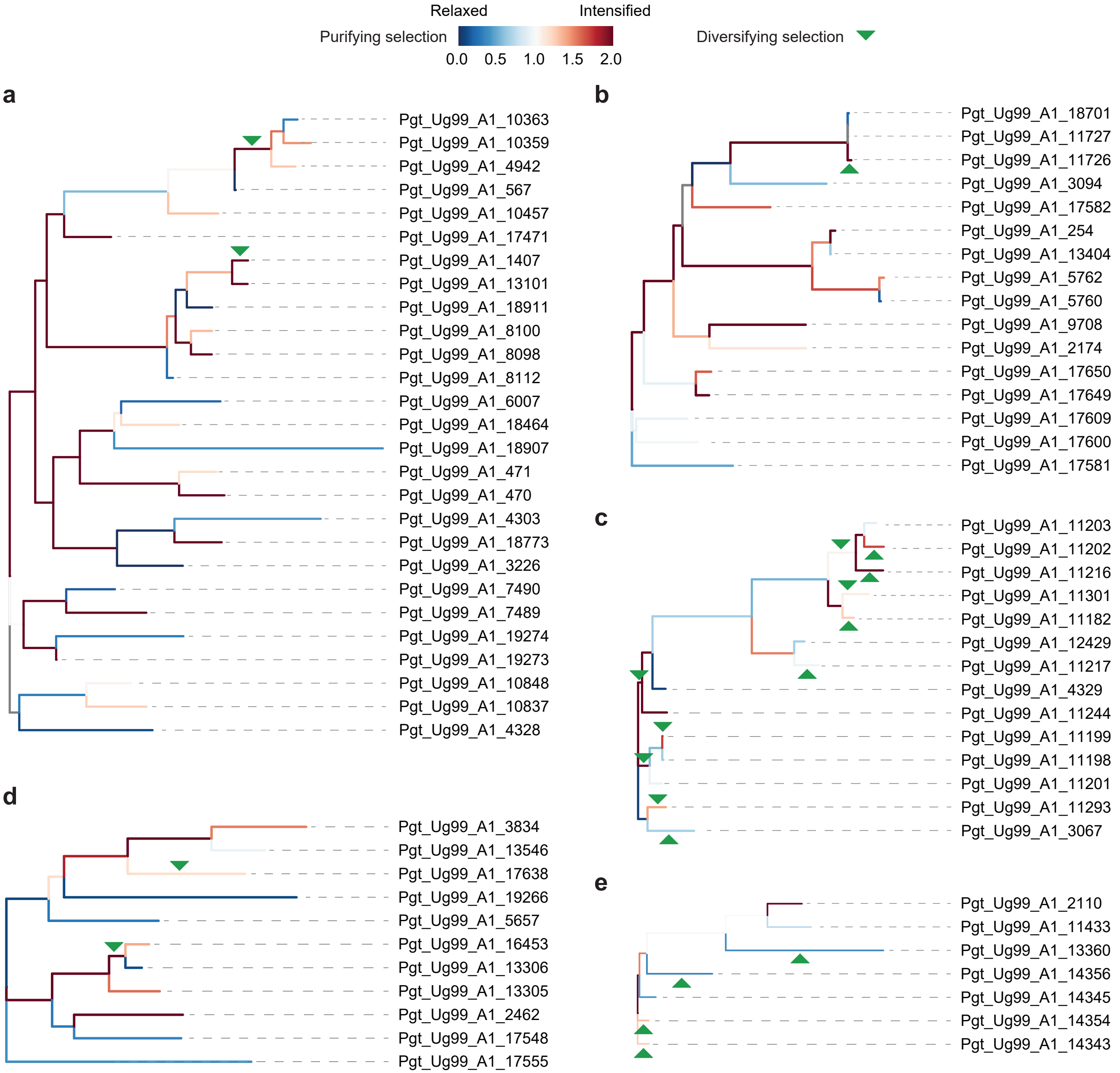

Supplementary Figure 12. Branch-level selection pressure on sequence-related subclusters of the hydrophobin-like effector group (Cluster 25).

The intensity of purifying selection on the terminal branches (test branches) were compared to the rest (reference branches) with RELAX to determine whether the purifying selection was overall relaxed or intensified after the most recent duplications that led to the terminal leaves. The significance of relaxation or intensification is captured through a likelihood ratio test computed by RELAX. For visualization, the descriptive selection intensity parameter k was normalized using geometric means, after limiting the maximum value of k to 10. The normalized k values were mapped to the branches, with the maximum normalized value capped at 2. The branch-level diversifying selection was examined with aBSREL, and the branches under diversifying selection ($p < 0.05$) are indicated with green triangles. **a**, HHsearch subcluster 121 with 27 members. The relaxation of selection was detected among the test branches relative to the reference branches ($p = 0.043$). Two branches were under diversifying selection. **b**, HHsearch cluster 220 with 16 members. No relaxation or intensification of selection was detected he test branches relative to the reference branches ($p = 1.0$). One branch was under diversifying selection. **c**, HHsearch cluster 242 with 14 members. The relaxation of selection was detected among the test branches relative to the reference branches ($p = 0.0127$). Eleven branch was under diversifying selection. **d**, HHsearch cluster 300 with 11 members. The relaxation of selection was detected among the test branches relative to the reference branches ($p = 0.0060$). Two branch was under diversifying selection. **e**, HHsearch cluster 449 with 7 members. No relaxation or intensification of selection was detected he test branches relative to the reference branches ($p = 0.6768$). Four branch was under diversifying selection.

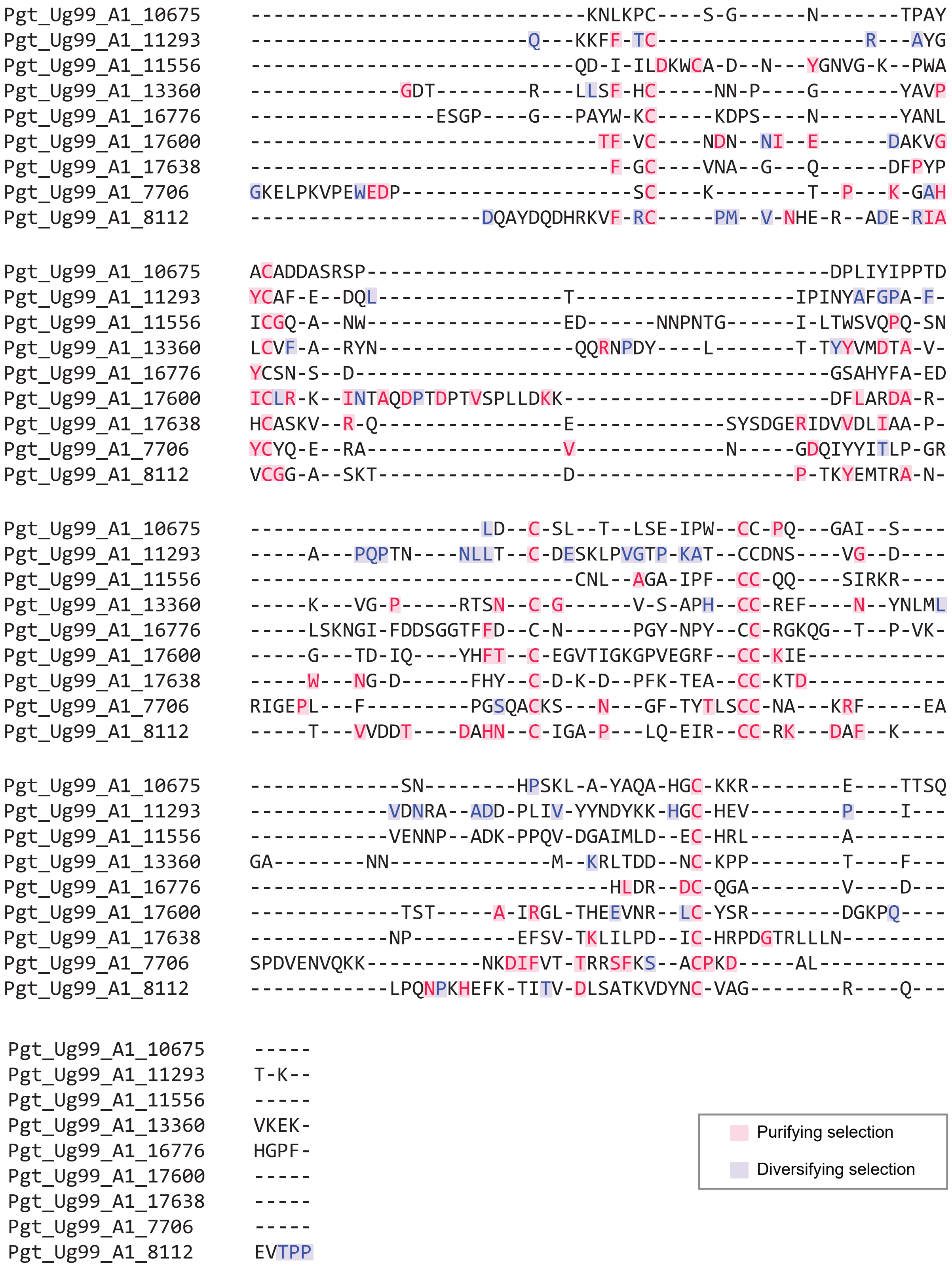

Supplementary Figure 13. Selection pressure mapped on the sequence alignment of the hydrophobin-like effector group.

The alignment of representative sequences from nine sequence-related subclusters in Cluster 25 with five or members: Pgt_Ug99_A1_8112 (HHsearch cluster 121; 27 members), Pgt_Ug99_A1_17600 (HHsearch cluster 220; 16 members), Pgt_Ug99_A1_11293 (HHsearch cluster 242; 14 members), Pgt_Ug99_A1_17638 (HHsearch cluster 300; 11 members), Pgt_Ug99_A1_13360 (HHsearch cluster 449; 7 members), Pgt_Ug99_A1_16776 (HHsearch cluster 570; 5 members), Pgt_Ug99_A1_7706 (HHsearch cluster 579; 5 members), Pgt_Ug99_A1_11556 (HHsearch cluster 595; 5 members) and Pgt_Ug99_A1_10675 (HHsearch cluster 614; 5 members). These members had the highest pTM score in their sequence-related subclusters. The alignment was constructed with mTM-align by superposing the predicted structures of the nine members. For each subcluster, purifying selection was detected with FEL and diversifying selection with FEL and MEME from HyPhy. The diversifying selection may be episodic or pervasive.

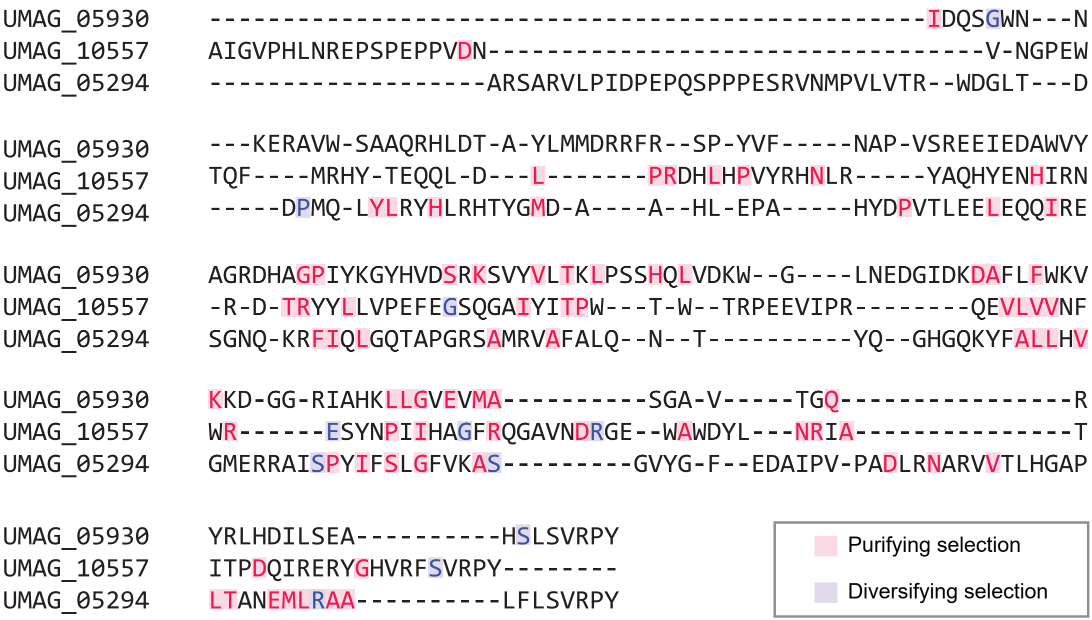

Supplementary Figure 14. Selection pressure on the Tin2-like effector group.

The alignment of representative sequences from three sequence-related subclusters in Cluster 96 with five or more members: UMAG_05930 (HHsearch cluster 380; 9 members), UMAG_10557 (HHsearch cluster 510; 6 members) and UMAG_05294 (HHsearch cluster 574, 5 members). These representative sequences have the highest pTM score in each subcluster. The alignment was constructed with mTM-align by superposing the predicted structures of the three members. For each subcluster, purifying selection was detected with FEL and diversifying selection with FEL and MEME from HyPhy. The diversifying selection may be episodic or pervasive. The sites under selection pressure were colored.

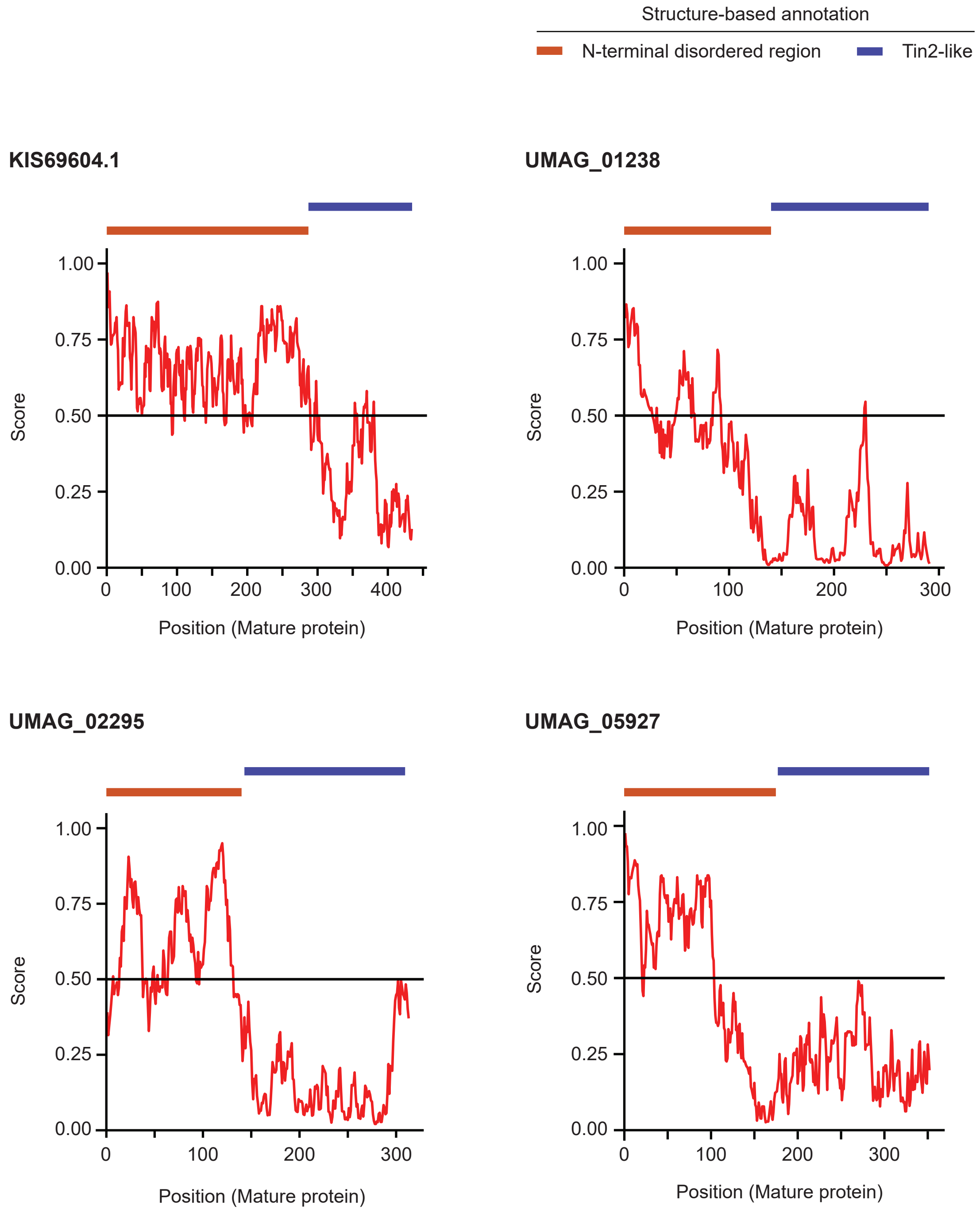

Supplementary Figure 15. The prediction of the disordered regions for the Tin2 fusion proteins.

IUPred2A was used to predict the intrinsically disordered regions for the Tin2 fusion proteins given in Figure 4. The structure-based disordered region and Tin2 fold are indicated with orange and blue bars on top of the plot. The score > 0.50 was used to determine the intrinsically disordered regions.

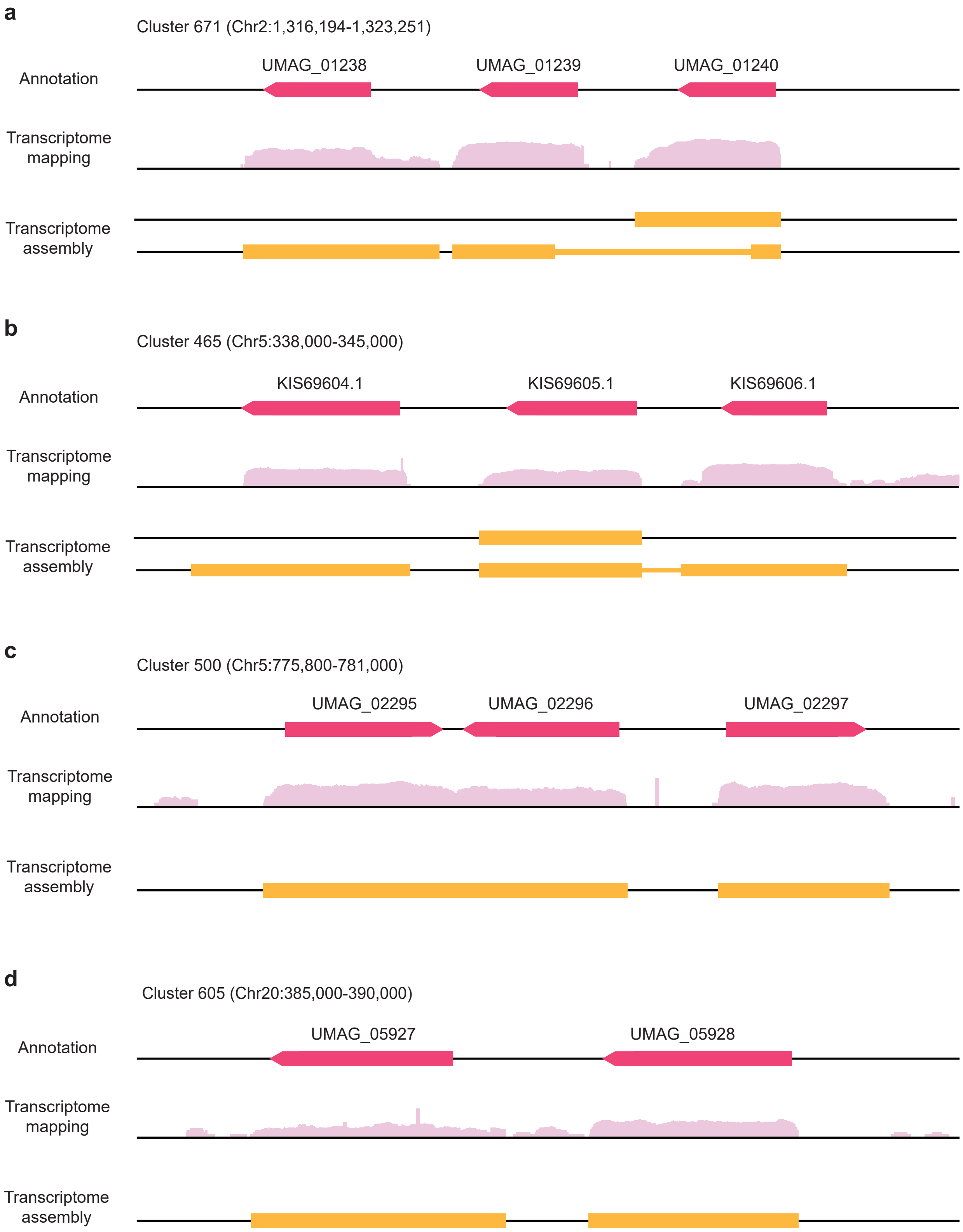

Supplementary Figure 16. Transcriptomic mapping and assembly results for the Tin2 fusion proteins.

A subset of publicly available transcriptomic data was aligned or assembled to examine the annotation quality of the Tin2 fusion proteins. The results are displayed for some Tin2 fusion proteins that exist in proximity.

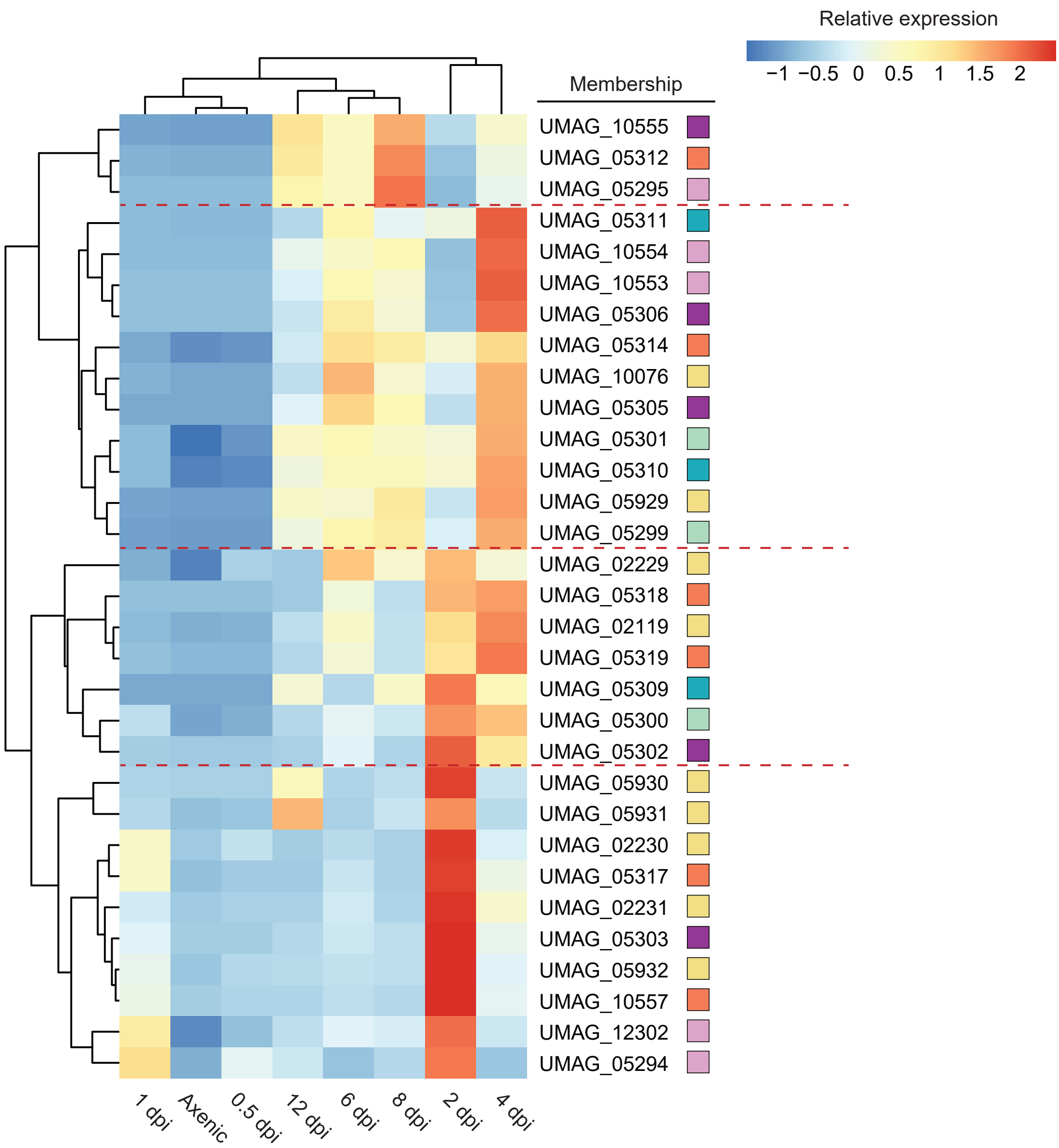

Supplementary Figure 17. Hierarchical clustering of the core Tin2-like cluster (Cluster 96).

The expression profiles of the Cluster 96 members were clustered with hierachical clustering. The membership of each sequence is indicated with colored boxes. The colors are the same as in Figure 4.
