# Supplementary material for: Prediction of effector protein structures from fungal phytopathogens enables evolutionary analyses
Source: Nat Microbiol. 2023 Jan 5;8(1):174–87. doi: 10.1038/s41564-022-01287-6 (PMC9816061; doi:10.1038/s41564-022-01287-6)
Supplement: Supplementary file 2 — Reporting Summary [file 41564_2022_1287_MOESM2_ESM.pdf]

nature portfolio

Corresponding author(s): Ksenia Krasileva

Last updated by author(s): Nov 8, 2022

Reporting Summary

Nature Portfolio wishes to improve the reproducibility of the work that we publish. This form provides structure for consistency and transparency in reporting. For further information on Nature Portfolio policies, see our Editorial Policies and the Editorial Policy Checklist.

Statistics

For all statistical analyses, confirm that the following items are present in the figure legend, table legend, main text, or Methods section.

n/a	Confirmed	
☐	☒	The exact sample size (*n*) for each experimental group/condition, given as a discrete number and unit of measurement
☒	☐	A statement on whether measurements were taken from distinct samples or whether the same sample was measured repeatedly
☐	☒	The statistical test(s) used AND whether they are one- or two-sided<br>*Only common tests should be described solely by name; describe more complex techniques in the Methods section.*
☒	☐	A description of all covariates tested
☒	☐	A description of any assumptions or corrections, such as tests of normality and adjustment for multiple comparisons
☐	☒	A full description of the statistical parameters including central tendency (e.g. means) or other basic estimates (e.g. regression coefficient) AND variation (e.g. standard deviation) or associated estimates of uncertainty (e.g. confidence intervals)
☐	☒	For null hypothesis testing, the test statistic (e.g. *F*, *t*, *r*) with confidence intervals, effect sizes, degrees of freedom and *P* value noted<br>*Give P values as exact values whenever suitable.*
☒	☐	For Bayesian analysis, information on the choice of priors and Markov chain Monte Carlo settings
☒	☐	For hierarchical and complex designs, identification of the appropriate level for tests and full reporting of outcomes
☒	☐	Estimates of effect sizes (e.g. Cohen's *d*, Pearson's *r*), indicating how they were calculated

Our web collection on statistics for biologists contains articles on many of the points above.

Software and code

Policy information about availability of computer code

Data collection	Secretome prediction: SignalP v3.0, InterProscan v5.30-69.0, and TMHMM v2.0<br>Structure prediction: AlphaFold v2.0.0, and HHsuite (HHfilter) v3.3.0<br>Functional and structural annotation: Gene 3D v4.3.0, PFAM v33.1, Superfamily v1.75, SCOPe v2.07, CATH v4.3.0, PDB (09/02/2021), Rupee, and IUPred2A
Data analysis	Network analyses: BLASTP v2.7.1, HHsuite (HHblits, HHsearch and HHfilter) v3.3.0, TM-align, MCL v14-137, networkx v2.2, and custom scripts (10.5281/zenodo.6480453)<br>Evolutionary analyses: FastTree v2.1.11, HyPhy v2.5.41, FEL v2.1, MEME v3.0, aBSREL v2.3, RELAX v3.1.1 and HMMER (hmmalign) v3.1b2

For manuscripts utilizing custom algorithms or software that are central to the research but not yet described in published literature, software must be made available to editors and reviewers. We strongly encourage code deposition in a community repository (e.g. GitHub). See the Nature Portfolio guidelines for submitting code & software for further information.

Data

Policy information about availability of data

All manuscripts must include a data availability statement. This statement should provide the following information, where applicable:

- Accession codes, unique identifiers, or web links for publicly available datasets
- A description of any restrictions on data availability
- For clinical datasets or third party data, please ensure that the statement adheres to our policy

Sequence data collection: MycoCosm from the Joint Genome Institute (https://mycocosm.jgi.doe.gov/mycocosm/home) and Ensembl Fungi (http://fungi.ensembl.org/index.html)
Structural data collection: Protein Data Bank (https://www.rcsb.org/; e.g. 7MQQ (AvrSr50 (QCMJC) and 7XC2 (AvrSr35))
Effector data collection: the Pathogen-Host Interactions database (PHI-base)
Datasets and scripts from this study: 10.5281/zenodo.6480453.

Human research participants

Policy information about studies involving human research participants and Sex and Gender in Research.

Reporting on sex and gender	N/A
Population characteristics	N/A
Recruitment	N/A
Ethics oversight	N/A

Note that full information on the approval of the study protocol must also be provided in the manuscript.

Field-specific reporting

Please select the one below that is the best fit for your research. If you are not sure, read the appropriate sections before making your selection.

☒ Life sciences ☐ Behavioural & social sciences ☐ Ecological, evolutionary & environmental sciences

For a reference copy of the document with all sections, see nature.com/documents/nr-reporting-summary-flat.pdf

Life sciences study design

All studies must disclose on these points even when the disclosure is negative.

Sample size	Species size determination: the species was selected based on the agricultural importance of fungal phytopathogens provided in Dean et al., (2012). A putative saprotrophic, non-phytopathogenic species was added for each order or subdivision as a control. Oomycete Phytophthora infestans as an outgroup.<br><br>Secretome size determination: SignalP v3.0 was used to to identify secreted proteins from the proteomes of the species.he candidates were excluded if their predicted signal peptides overlapped with PFAM domains annotated with InterProscan v5.30-69.0 over 10 or more amino acids, or if their mature proteins contained any transmembrane helices detected with TMHMM v2.0.
Data exclusions	Secretome determination: The prediction of the N-terminal signal peptides on the region in which PFAM domains are present was indicative of false prediction of the signal peptides. The presence of transmembrance helices in the mature proteins was suggestive of the localization of the mature proteins in or at the fungal cell wall.<br><br>Protein structure prediction: Any short mature proteins smaller than 15 amino acids in length were removed, as they would be unfordable peptide stretches. Any large mature proteins larger than 860 amino acids in length were also not modeled due to the limitation of our GPUs. These proteins represent a very small fraction of the data. These excluded proteins are only a very small portion of the secreted proteins.
Replication	Replication was not performed as our data were not random samples
Randomization	Randomization was not performed as our data were not random samples.
Blinding	Blinding was not performed as our data were not random samples.

Reporting for specific materials, systems and methods

We require information from authors about some types of materials, experimental systems and methods used in many studies. Here, indicate whether each material, system or method listed is relevant to your study. If you are not sure if a list item applies to your research, read the appropriate section before selecting a response.

Materials & experimental systems

n/a	Involved in the study
☒	☐ Antibodies
☒	☐ Eukaryotic cell lines
☒	☐ Palaeontology and archaeology
☒	☐ Animals and other organisms
☒	☐ Clinical data
☒	☐ Dual use research of concern

Methods

n/a	Involved in the study
☒	☐ ChIP-seq
☒	☐ Flow cytometry
☒	☐ MRI-based neuroimaging
